# Sustained effects of neurofeedback in ADHD: a systematic review and meta-analysis

**DOI:** 10.1007/s00787-018-1121-4

**Published:** 2018-02-14

**Authors:** Jessica Van Doren, Martijn Arns, Hartmut Heinrich, Madelon A. Vollebregt, Ute Strehl, Sandra K. Loo

**Affiliations:** 1 Department of Child and Adolescent Mental Health, University Hospital Erlangen, Erlangen, Germany; 2 Department of Experimental Psychology, Utrecht University, Utrecht, The Netherlands; 3 neuroCare Group, Munich, Germany; 4 Research Institute Brainclinics, Bijleveldsingel 34, 6524 AD Nijmegen, The Netherlands; 5 kbo-Heckscher-Klinikum, Munich, Germany; 6 Department of Cognitive Neuroscience, Donders Institute for Brain, Cognition and Behaviour, Radboud University Medical Centre, Nijmegen, The Netherlands; 7 Institute for Medical Psychology, University of Tuebingen, Tuebingen, Germany; 8 Department of Psychiatry and Biobehavioral Science, David Geffen School of Medicine, University of California, Los Angeles, USA

**Keywords:** Neurofeedback, EEG biofeedback, ADHD, Meta-analysis, Sustainability, Follow-up

## Abstract

Neurofeedback (NF) has gained increasing interest in the treatment of attention-deficit/hyperactivity disorder (ADHD). Given learning principles underlie NF, lasting clinical treatment effects may be expected. This systematic review and meta-analysis addresses the sustainability of neurofeedback and control treatment effects by considering randomized controlled studies that conducted follow-up (FU; 2–12 months) assessments among children with ADHD. PubMed and Scopus databases were searched through November 2017. Within-group and between-group standardized mean differences (SMD) of parent behavior ratings were calculated and analyzed. Ten studies met inclusion criteria (NF: ten studies, *N* = 256; control: nine studies, *N* = 250). Within-group NF effects on inattention were of medium effect size (ES) (SMD = 0.64) at post-treatment and increased to a large ES (SMD = 0.80) at FU. Regarding hyperactivity/impulsivity, NF ES were medium at post-treatment (SMD = 0.50) and FU (SMD = 0.61). Non-active control conditions yielded a small significant ES on inattention at post-treatment (SMD = 0.28) but no significant ES at FU. Active treatments (mainly methylphenidate), had large ES for inattention (post: SMD = 1.08; FU: SMD = 1.06) and medium ES for hyperactivity/impulsivity (post: SMD = 0.74; FU: SMD = 0.67). Between-group analyses also revealed an advantage of NF over non-active controls [inattention (post: SMD = 0.38; FU: SMD = 0.57); hyperactivity–impulsivity (post: SMD = 0.25; FU: SMD = 0.39)], and favored active controls for inattention only at pre-post (SMD = − 0.44). Compared to non-active control treatments, NF appears to have more durable treatment effects, for at least 6 months following treatment. More studies are needed for a properly powered comparison of follow-up effects between NF and active treatments and to further control for non-specific effects.

**Electronic supplementary material:**

The online version of this article (10.1007/s00787-018-1121-4) contains supplementary material, which is available to authorized users.

## Introduction

Clinical guidelines for attention-deficit/hyperactivity disorder (ADHD) recommend multimodal treatment approaches, with current evidence suggesting that medication, including methylphenidate and various amphetamine formulations, in conjunction with psychosocial treatment are most effective in the short-term [1]. Medication treatments have large effect size in the acute treatment of ADHD [2] and, when combined with psychosocial treatments, large effects up to 2 years of treatment were observed [3, 4]. Nevertheless, it is widely accepted that further treatments with long-lasting effects have to be developed and evaluated.

Over the last decade, an increasing number of studies investigating non-pharmacological treatments have been published. Neurofeedback (NF), which aims at improving self-regulation of brain activity (most often the electroencephalogram, EEG) using a brain–computer interface, has gained popularity [5]. A promising aspect of neurofeedback is that it may rely on procedural learning, thereby potentially allowing lasting effects and thus longer clinical benefit after completion of neurofeedback treatment. In their review, Arns and Kenemans [6] found that the clinical effects of neurofeedback were maintained across 6 and 24-month follow-up periods, with a trend for larger symptom decreases for hyperactivity/impulsivity after 24 months than after 6 months, albeit only based on two randomized studies at 6 months and only one at the 24-month follow-up, thus limiting the generalizability of the findings. A systematic review and meta-analysis that assess the sustainability of clinical effects of NF studies is, therefore, desirable.

In recent years, several randomized control studies (RCTs) and meta-analyses have been published on the efficacy of neurofeedback for children with ADHD, overall with mixed results and interpretations [7–11]. Regarding RCTs published over the last decade, one major issue is the lack of standardization of neurofeedback protocols and implementations. Neurofeedback treatments using theta/beta, slow cortical potential (SCP), or sensorimotor-rhythm (SMR) protocols have been well studied and can be seen as ‘standard’ neurofeedback treatments (for review and discussion see: Arns et al. [5] and Figure S-2 in Supplementary Material). These ‘standard’ protocols have been selected as the primary protocols for NF research based on findings that children with ADHD have specific deficits in comparison with healthy controls: e.g. increased theta/beta ratios in subgroups of ADHD patients hypothesized to be related to inattention [12]; decreased contingent negative variation amplitudes (targeted by SCP training) [13]; addressing hyperkinetic behavior by means of training sensorimotor rhythm [14]. Application of these standard neurofeedback protocols in ADHD have most consistently resulted in clinical benefit in children with ADHD, whereas application of other neurofeedback protocols has yielded more variable and mixed results [5, 8]. A second issue with currently available neurofeedback RCTs is that several studies deviated from their initial clinical trials register, with samples ranging from 34% [15] to 60% [16, 17] smaller than their preregistered sample size. This raises the likelihood of over-interpreting results from these studies, which are insufficiently powered. This issue is addressed using meta-analyses since effects are combined across studies, resulting in increased statistical power.

A third issue concerns the specificity of NF treatment effects. While NF has been shown to be beneficial for the treatment of ADHD symptoms, it remains a debate whether behavioral improvements are the result of specific aspects of the NF treatment such as the style of training, or active learning of control over their brain state that is then generalized to daily life or whether non-specific treatment effects such as unconditional positive regard of the therapist, positive expectation of change, or repeated practice of sitting at a computer for increasing lengths of time leads to behavioral change. To control these non-specific or placebo effects, double-blind placebo-controlled studies are often requested—comparable to what is considered as gold standard in drug research. However, in regard to NF, there are methodical and ethical issues to consider which have led to the development of control conditions such as cognitive training or EMG biofeedback and assessments for placebo factors via evaluation scales [18, 19]. Though it is known that placebo effects may last over longer periods [20], it seems unlikely that they grow larger over time. Hence focusing on longer-term outcomes of NF may also help to clarify the placebo/specific vs. non-specific issue.

Two recent meta-analyses on the acute efficacy of neurofeedback for children with ADHD published by the European ADHD Guidelines Group (EAGG) have used the interesting concept of most-proximal (e.g. least blinded, often parent ratings) versus probably blinded measures (most often teacher ratings), assuming that the probably blinded measures (i.e., teacher ratings) are less susceptible to expectation/non-specific effects and, therefore, more valid [8, 11]. However, this approach has limitations. For example, parent–teacher correlations on behavior rating scales are only modestly correlated (ranging from 0.23 to 0.49) [21, 22], suggesting different aspects of the disorder may be detected by different raters or in different settings. Furthermore, in a large candidate gene study, parent-rated hyperactive–impulsive behaviors were significantly associated with candidate gene pathways whereas teacher ratings were not [23]. Additionally, teacher-ratings are sensitive to effects of methylphenidate [24], which could possibly skew the interpretation of studies that randomize ADHD treatments against a methylphenidate control, when primarily relying on teacher reports. Finally, for investigating long-term effects, it may not be advisable to rely on teacher ratings, since the child may have more than one teacher over time, potentially compromising the reliability of the rating.

A second limitation of the aforementioned meta-analyses is the use of between-group effect sizes, which is a good practice for compiling results from studies that used similar designs and control groups; comparing for example, psychostimulants to placebo. However, the utility of this method for studying results across neurofeedback studies, with various kinds of control groups (ranging from waiting lists, cognitive training to medication), is more challenging. Therefore, while between-group effect sizes are useful for controlling for non-specific effects of treatment, they can miss clinical effects of neurofeedback that are masked by the active or semi-active control conditions, thus warranting the use of a within-group effect-size approach, for example as used by Arns et al. [7], or separately analyze the results for active vs. semi-active control groups.

To address the above concerns, we have conducted a systematic review and meta-analysis of the post-treatment follow-up period of randomized EEG NF studies among children and adolescents with ADHD. Methodologically, we (1) used within-group effect sizes to address the issue of different control groups; (2) used between-group effect sizes to control for non-specific effects of treatment; (3) applied a meta-analytical approach to address the issue of underpowered studies; and (4) focused on the sustainability of treatment effects by looking specifically at the follow-up (FU) period (i.e., pre-FU, post-FU time points), which will provide information regarding the plausibility of sustainable NF effects, relative to other treatments, in ADHD.

## Method

### Study selection

The protocol of this meta-analysis was not preregistered. A literature search was conducted up to 29th of November 2017 via PubMed and Scopus by author JVD, looking for studies investigating Neurofeedback or EEG Biofeedback in ADHD using combinations of the following keywords: ‘Neurofeedback’, ‘EEG Biofeedback’, ‘Neurotherapy’, ‘SCP’ OR ‘Slow Cortical Potentials’ AND ‘ADHD’, ‘ADD’, ‘Attention Deficit’ OR ‘Attention Deficit Hyperactivity Disorder’. Furthermore, prior meta-analyses and systematic review reference lists were inspected for potentially missed studies [7, 8, 10, 11]. After exclusion of duplicate publications, abstracts were screened for inclusion criteria first by author HH and then by a research assistant to prevent missing studies. Studies that remained of interest were then screened based on their full text by JVD. Inclusion criteria were: (1) randomized controlled EEG neurofeedback trials published in peer-reviewed journals; (2) primary diagnosis of ADHD; (3) mean child age < 18 years old; (4) available data at a follow-up (FU) time point for 2 to 12 months post-treatment; (5) standardized mean and standard deviations (SD) for all three assessments (pre, post, and FU) for at least one of the following domains had to be available: inattention, hyperactivity, or hyperactivity/impulsivity ratings from a DSM-IV/5-based rating scale (these values were taken based on availability with parental ratings taking priority, then self-ratings and lastly teacher ratings); (6) publication available in English; (7) total study sample larger than *N* = 10; 8) less than 50% of participants began or stopped taking medication between post and FU assessments.

In most of the studies, ‘standard’ neurofeedback protocols [5] were used, i.e., theta/beta and theta/SMR (sensorimotor rhythm) training (defined as a down-training of theta and up-training of beta and SMR, respectively) and slow cortical potential training (SCP) training (addressing modulation of positive and negative SCPs), for details see Table 1 and Supplementary Figure S-2. Exceptions were the studies of Arnold et al. [25] and Bink et al. [26] which targeted more EEG frequency bands (theta, alpha, SMR, and beta), and a sensitivity analysis was conducted to separately assess the effects for standard protocols. When the means and SDs from a given study were not available, or it was unclear if planned follow-up measurements were published, this information was requested via email from the authors. If authors did not respond or did not provide the missing information, and if there was not sufficient information available based on the publication, the study was excluded from the meta-analysis. Studies were additionally screened for duplicate data based on author, publication year, participant numbers and trial registration number (if available). When possible duplicate data was found, the authors were contacted to clarify whether the data sets were independent.

**Table 1:** Characteristics of included studies

Study or subgroup	Year	*N* (FU)	Age	Treatment	FU (months)	Assessment instrument	Medicated/ total *N*	Medication dosage^a^
Neurofeedback								
Heinrich et al. [36]	2004	13	11.1 ± 1.6	SCP (Cz); 25 sessions of 50 min	3	FBB-HKS	6/13	No change
Gevensleben et al. [52]	2010	38	9.9 ± 1.3	SCP + theta (4–8 Hz)/beta (13–20 Hz); Cz; 36 sessions of 50 min	6	FBB-HKS	0/38	No change
Arnold et al. [25]	2013	25	9.0 ± 1.5	Theta/alpha**↓**; beta/SMR**↑**; Cz; 40 sessions of 45 min	2	Conners DSM	7/25	Increased
Li et al. [39]	2013	31	10.8 ± 2.6	MPH (pre) + NF; theta (4–8 Hz)/SMR (12–15 Hz); electrode NR;40 sessions of 25–30 min	6	ADHD RS-IV	31/31	Decreased
Meisel et al. [37]	2013	12	9.5 ± 1.8	Theta (4–7 Hz)/beta 15–20 Hz); Cz or FCz; 40 session of 30 min	2	ADHD RS-IV	2/12	Increased
Steiner et al. [34]	2014	34	8.4 ± 1.1	Theta (4–8 Hz)/SMR (12–15 Hz); electrode NR; 40 sessions of 45 min	6	Conners	27/34	Maintained
Christiansen^b^	2014	18	8.7 ± 1.4	SCP; Cz; 30 sessions of 50 min	6	Conners DSM	1/18	Decreased
Bink et al. [26]	2016	41	15.8 ± 3.3	TAU + NF; theta/alpha (4–7, 8–11 Hz) ↓, SMR (13–15 Hz) ↑, beta/gamma (22–36 Hz) ↓; Cz; 40 sessions of 30 min	12	ADHD RS-IV (self-report)	19/41	NR
Duric et al. [38]	2017	24	11.3 ± 2.8	Theta (4–7 Hz)/beta (16–20 Hz); Cz; 30 sessions of 40 min	6	Barkley	0/24	No change
Gelade et al. [35]	2017	20	9.8 ± 1.9	Theta (4–8 Hz)/beta (13–20 Hz); Cz; 30 sessions of 45 min	6	SWAN	0/20	No change
Control conditions								
Gevensleben et al. [52]	2010	23	9.4 ± 1.1	Attention training; 36 sessions of 50 min	6	FBB-HKS	0/23	No change
Arnold et al. [25]	2013	11	8.7 ± 2.1	Sham neurofeedback; 40 sessions of 45 min	2	Conners DSM	0/11	No change
Li et al. [39]	2013	29	10.4 ± 2.9	MPH (pre) + non-feedback attention training 40 sessions of 25–30 min	6	ADHD RS-IV	29/29	Maintained
Meisel et al. [37]	2013	11	8.9 ± 1.5	MPH (inferior dosage: 1 mg/kg/day)	2	ADHD RS-IV	11/11	No change
Steiner et al. [34] CT	2014	34	8.9 ± 1.0	Cognitive training; 40 sessions of 45 min	6	Conners	14/34	Increased
Steiner et al. [34] WL	2014	36	8.4 ± 1.1	Wait list	6	Conners	20/36	Increased
Christiansen^b^	2014	21	8.9 ± 1.2	Self-management; 30 sessions of 50 min	6	Conners DSM	6/21	Increased
Bink et al. [26]	2016	19	16.2 ± 3.4	TAU	12	ADHD RS-IV (self-report)	12/19	NR
Duric et al. [38]	2017	28	10.8 ± 2.4	MPH (1 mg/kg/day; range: 20 to 60 mg)	6	Barkley	29/29	No change
Gelade et al. [35] MPH	2017	21	9.0 ± 1.2	MPH (5–20 mg daily)	6	SWAN	21/21	NR
Gelade et al. [35] PA	2017	17	9.6 ± 1.8	Physical activity training; 28 sessions of 30 min	6	SWAN	0/17	No change

For neurofeedback (NF) protocols, frequency bands and feedback electrodes are listed. Parent ratings were considered (except for Bink et al. [26] which used self-reports)

*SCP* slow cortical potential, *SMR* sensorimotor rhythm, *MPH* methylphenidate, *PA* physical activity, *FBB-HKS* German ADHD rating scale for parents, *ADHD RS-IV* ADHD Rating Scale-IV, *Conners* Conners questionnaire subscale, *Conners DSM* DSM subscale within the Conners questionnaire; *SWAN* strengths and weaknesses of ADHD symptoms and Normal Behavior Scale, *NR* not reported

^a^For more study details see Table S-4

^b^Unpublished data from Christiansen et al. [40]

### Data extraction/outcome measures

Data were first extracted by JVD and checked by HH. The following pre-, post- and FU-assessment measures were extracted from the included studies:

1. Demographic and clinical data: age (mean and standard deviation), medication use, ADHD subtype.
2. Experimental procedure: NF method, control method, feedback electrode, average number of sessions, session length.
3. Outcome measures:
  Symptom domains: Assessed from parent report with a validated ADHD rating scale (e.g., DSM-IV rating scale [27], Conners [28], Barkley [29], FBB-HKS [30], SWAN [31])
  (i) Inattention
  (ii) Hyperactivity/impulsivity (if no combined measure was available, the hyperactivity score was used).

These measures were used as treatment endpoints at post-treatment and FU relative to baseline values. Additionally, the change from post-treatment to FU was assessed to determine if changes occurred after the treatment had stopped.

### Meta-analysis

A random effects model (due to inherent heterogeneity between studies) and the inverse variance statistical method was used to calculate the standardized mean difference (SMD), 95% confidence intervals, and *χ*^2^ statistic using RevMan version 5.3 [32]. Within-group and between-group analyses were conducted for the following time points using the means and standard deviations provided in the papers or by the authors: (1) pre- and post-treatment; (2) pre-treatment and follow-up; (3) post-treatment and follow-up. Within-group analyses used the values as presented in the papers (no additional calculation was necessary). Between-group means were calculated by subtracting the mean of the second time point from the mean of the first time point (ex. pre-treatment minus post-treatment, pre-treatment minus FU-treatment, post-treatment minus FU-treatment). The standard deviation of the first point was used for analysis.

Although active treatment effects are not the main focus of this paper, the control conditions were analyzed as a whole and assessed using two sub-analyses (non-active and active control conditions) to provide a frame of reference for the effects of neurofeedback against different type of controls. Due to the diversity of the control conditions, we chose to separate them as ‘active’ (proven to have a clinical effect in the treatment of ADHD: methylphenidate and self-management training) and ‘non-active’ (all conditions that do not classify as active). For a more detailed explanation of these groupings see Table S-2 in the supplement. See Tables S-5 and S-6 for values used. When the *χ*^2^ statistic of a sample (*Q*_t_) was significant (*p* < 0.05)—indicating that the variance among effect sizes is greater than expected by sampling error—studies were assessed for possible heterogeneity causes and the resulting studies were omitted from the meta-analysis, for example based on the type of treatment (separating active and non-active conditions in the control groups). Active treatments were defined as medication or psychotherapy (self-management) that was started systematically after pre-treatment assessment. Additionally, a sensitivity analysis was conducted including only studies that used standard NF protocols (theta/beta, theta/SMR, or SCP). To assess publication bias, MetaWin version 2.1 [33] was used to calculate the fail-safe number (Rosenthal’s method: *a* < 0.05).

## Results

A total of ten studies met inclusion criteria for at least one of the parameters and conditions, resulting in ten studies in the NF arm and nine studies in the control arm (two control studies had two control groups [34, 35]). See Figure S-1 for the Preferred Reporting Items for Systematic Reviews and Meta-Analyses (PRISMA) inclusion flow diagram and Table 1 for characteristics of included studies. The PRISMA checklist is available in the supplementary material (Table S-1). The supplementary material has a complete list of inclusions (Table S-4) and exclusions (Table S-3). Included studies resulted in a total of 506 participants with ADHD (256 neurofeedback, 250 control). Follow-up time periods were 2 months (*K* = 2), 3 months (*K* = 1), 6 months (*K* = 6) and 12 months (*K* = 1).

The control group from Heinrich et al. [36] was excluded because most of the controls began psychotherapy between post and FU (personal communication with first author). The 2-month FU time point from the Meisel et al. [37] study was included instead of the 6-month FU because over 50% of their NF participants began medication between post- and 6-month FU measurements while only two participants started taking medication between post and 2-month FU. The combined NF + MPH arm of Duric et al. [38] was excluded since the NF treatment was accompanied by another active treatment, which did not allow differentiation of treatment effects; this differed from other studies in which NF children could receive medication if they had been already medicated prior to study participation, ensuring that the baseline measurements already included medication effects. The supplementary analysis was used from Gelade et al. [35] instead of the primary analysis to account for participant drop-out and medications change. For the majority of the studies, data were available for participants who completed assessments at all three time points (pre-, post-, FU) with the exception of Li et al. [39], in which drop-out from baseline to FU were 3.13% (*N* = 1) for the NF group and 9.38% (*N* = 3) for the control group.

Regarding medication change for included studies, the number of participants who began or stopped taking medication did not change over time for most of the studies, with the exception of four [25, 26, 37, 40]. In summary: of the total NF participants three stopped taking medication [pre-post (*N* = 2), post-FU (*N* = 1)], while nine began taking medication [pre-post (*N* = 1), post-FU (*N* = 8)]; for the control group only one participant stopped taking medication from post to FU time point. Dosage was allowed to be changed in five studies (see Table 1); however, dosage change was variable with some groups maintaining (one NF, one control), decreasing (two NF) or increasing (two NF, three control) dosage. See Table S-4 for details.

### Within-group analyses

#### Inattention

**Fig. 1:**
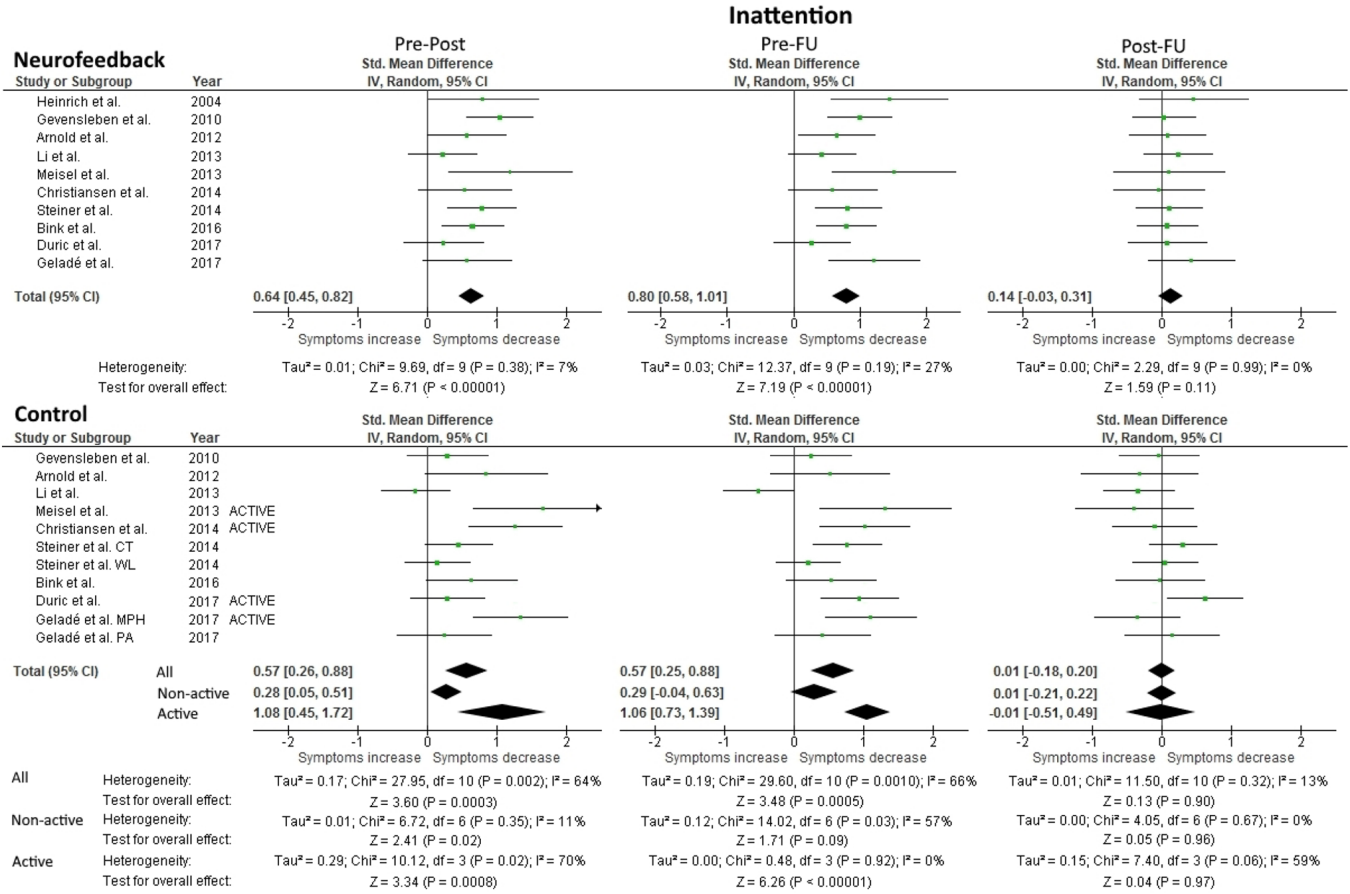
Forest Plot of within-group analysis for inattention parameter. Total standardized mean difference (SMD) with 95% confidence interval, overall effect, and heterogeneity are reported. Due to significant heterogeneity in the initial control analysis, additional analyses examining non-active and active controls separately were included. Pre-Post refers to the difference in means at pre- and post-measurement, and similarly for pre-FU and post-FU

Forest plots and results for inattention are presented in Fig. 1; Bar plots in Figure S-3. The test for heterogeneity was not significant for NF at pre-post (*χ*^2^ = 9.69, *df* = 9, n.s.) or pre-FU (*χ*^2^ = 12.37, *df* = 9, n.s.), while controls did display significant heterogeneity for both pre-post (*χ*^2^ = 27.95, *df* = 10, *p* < 0.05) and pre-FU (*χ*^2^ = 29.60, *df* = 10, *p* < 0.05) when all controls were included. When considering non-active controls only, heterogeneity was no longer significant for the pre-post measurement (*χ*^2^ = 6.72, *df* = 6, n.s.), but remained significant for the pre-FU measurement (*χ*^2^ = 14.02, *df* = 6, *p* < 0.05). When only active controls were included, pre-post heterogeneity remained significant (*χ*^2^ = 10.12, *df* = 3, *p* < 0.05) while pre-FU (*χ*^2^ = 0.48, *df* = 3, n.s.) was not significant. These results suggest that when heterogeneity is significant for controls, it will be useful to examine SMDs for active and non-active controls separately, which will be done for the remainder of the paper. This reduces the number of studies examined, however, and caution will be used when interpreting the results. Heterogeneity was non-significant for all groups for the post-FU measurement: NF (*χ*^2^ = 2.29, *df* = 9, n.s.); non-active (*χ*^2^ = 4.05, *df* = 6, n.s.); active (*χ*^2^ = 7.40, *df* = 3, n.s.).

NF yielded a significant medium effect size (SMD = 0.64; 95% CI 0.45, 0.82) for the change from the pre- to post-treatment measurements and a significant large effect size (SMD = 0.80; 95% CI 0.58, 1.01) for the change from pre- to FU measurement; however, post-treatment to FU was not significant (SMD = 0.14; 95% CI − 0.03, 0.31). For non-active controls, a small, significant effect for pre-post (SMD = 0.28; 95% CI 0.05, 0.51) was found but the small effect at pre-FU was no longer significant (SMD = 0.29; 95% CI − 0.04, 0.63). When looking at only active controls, there were large, significant effect sizes at both pre-post (significant heterogeneity) (SMD = 1.08; 95% CI 0.45, 1.72) and pre-FU (non-significant heterogeneity) (SMD = 1.06; 95% CI 0.73, 1.39). Post-treatment to FU was not significant for either control group. The fail-safe numbers for NF were: pre-post (156.0), pre-FU (190.7). For the control conditions, the fail-safe numbers were: 1. non-active controls: pre-post (11.2), pre-FU (1.1); 2. active controls: pre-post (13.5), pre-FU (55.2).

#### Hyperactivity/impulsivity

**Fig. 2:**
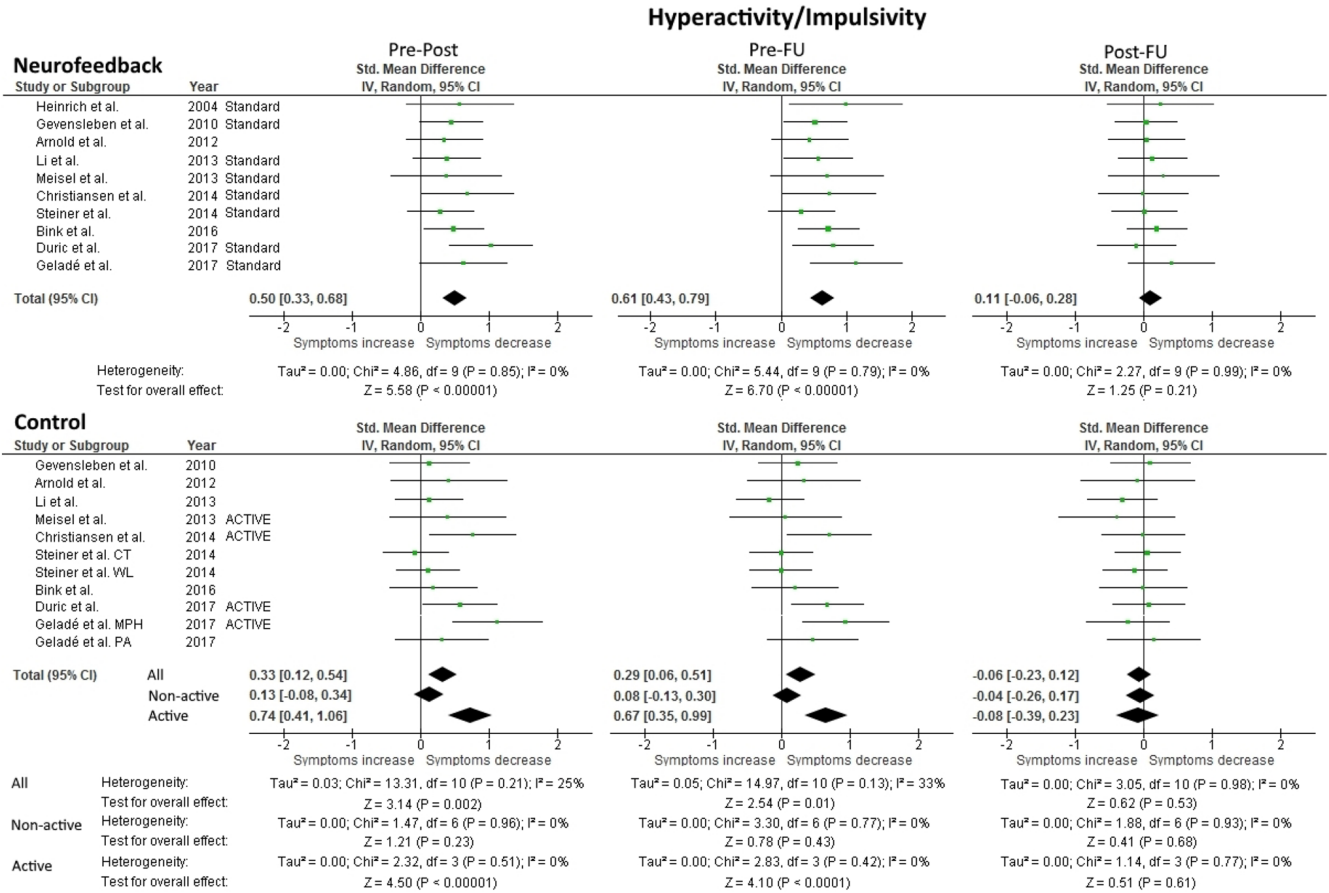
Forest Plot of within-group analysis for hyperactivity/impulsivity parameter. Total standardized mean difference (SMD) with 95% confidence interval, overall effect, and heterogeneity are reported. Analysis of the control condition separately for non-active and active controls was conducted for comparability to the inattention parameter analysis. Pre-Post refers to the difference in means at pre- and post-measurement, and similarly for pre-FU and post-FU

Forest plots and results for hyperactivity/impulsivity are presented in Fig. 2; Bar plots in Figure S-3. The test for heterogeneity was not significant for any of the hyperactivity/impulsivity measurements, indicating that the variance of SMD was not large enough to be attributed to sampling error only (see Fig. 2). For comparability to the inattention domain, the active and non-active control groups are reported separately.

For NF, a significant medium effect size (SMD = 0.50; 95% CI 0.33, 0.68) was found for the pre-post measurement and a medium effect size (SMD = 0.61; 95% CI 0.43, 0.79) for the pre-FU measurement; the post-FU measurement was not significant (SMD = 0.11; 95% CI − 0.06, 0.28).

Analysis of non-active control groups indicated that none of the measurements (pre-post, pre-FU, post-FU) were significant. When only active controls were considered, there were significant medium effect sizes for both pre-post (SMD = 0.74; 95% CI 0.41, 1.06) and pre-FU (SMD = 0.67; 95% CI 0.35, 0.99). For both control analyses, post-FU was not significant. The fail-safe numbers for NF were: pre-post (107.6), pre-FU (163.4). For the controls, the fail-safe numbers were: 1. non-active controls: pre-post (0), pre-FU (0); 2. active controls: pre-post (25), pre-FU (18.4).

### Between-group meta-analysis

**Fig. 3:**
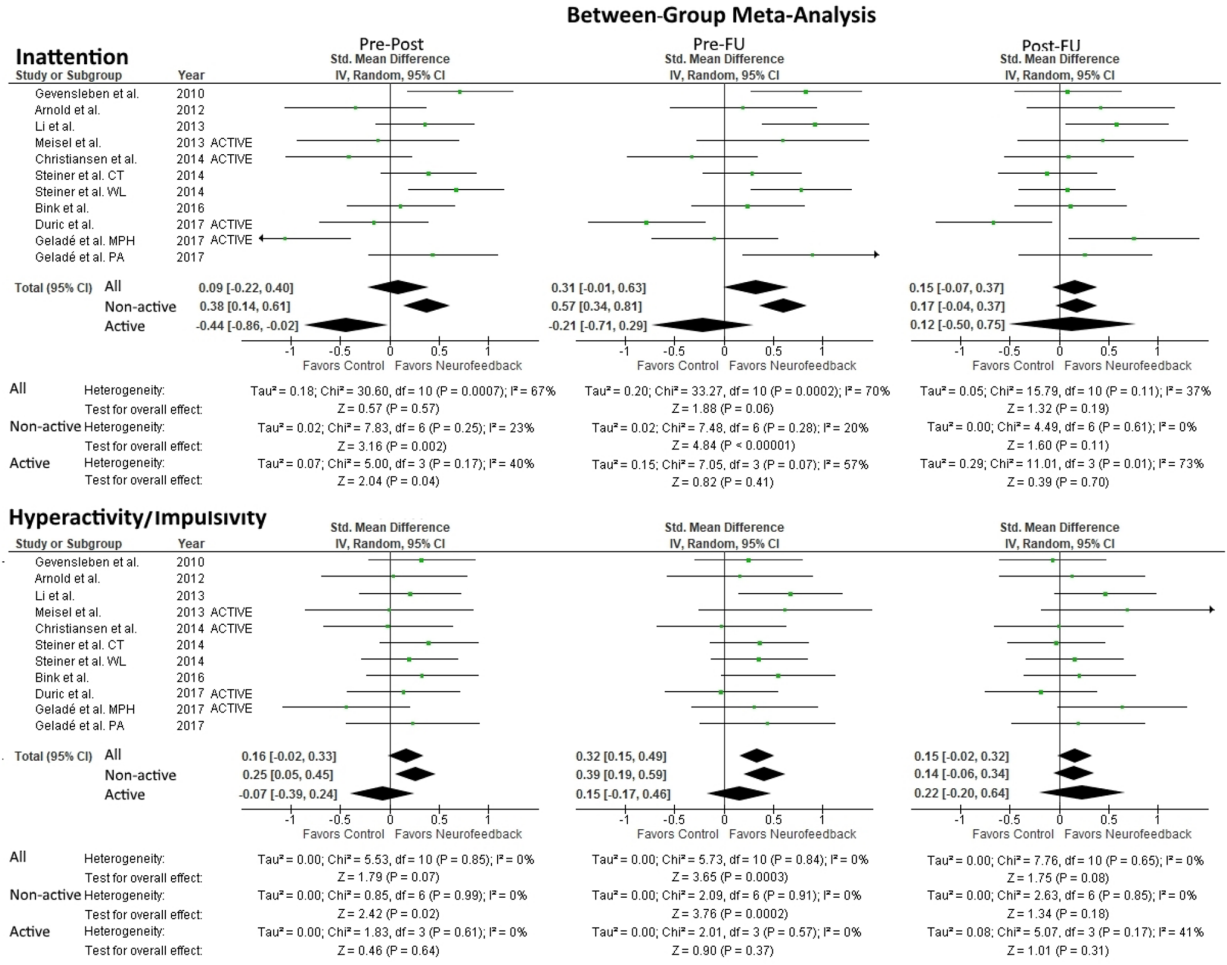
Forest Plot of between-group analysis for inattention and hyperactivity/impulsivity parameter. Total standardized mean difference (SMD) with 95% confidence interval, overall effect, and heterogeneity are reported. Analysis of the studies separately for non-active and active controls was conducted for comparability to the inattention parameter analysis. Pre-Post refers to the difference in means at pre- and post-measurement, and similarly for pre-FU and post-FU

Forest plots and results for inattention and hyperactivity/impulsivity are presented in Fig. 3.

#### Inattention

The test for heterogeneity was significant when including all studies at pre-post (*χ*^2^ = 30.60, *df* = 10, *p* < 0.05) and significant at pre-FU (*χ*^2^ = 33.27, *df* = 10, *p* < 0.001). Considering only trials with non-active control conditions, heterogeneity was not significant for the pre-post measurement (*χ*^2^ = 7.83, *df* = 6, n.s.) or the pre-FU measurement (*χ*^2^ = 7.48, *df* = 6, n.s.). For active controls, heterogeneity was not significant for pre-post (*χ*^2^ = 5.00, *df* = 3, n.s.) or for pre-FU (*χ*^2^ = 7.05, *df* = 3, n.s.).

When including only studies with non-active control conditions, a significant small effect size for pre-post (SMD = 0.38; 95% CI 0.14, 0.61) and a medium effect size for pre-FU (SMD = 0.57; 95% CI 0.34, 0.81) were observed favoring neurofeedback. When only active controls were included, a pre-post effect size favoring active controls was significant (SMD = − 0.44; 95% CI − 0.86, − 0.02) but at pre-FU it was no longer significant. Post-training to FU was not significant for either analysis.

#### Hyperactivity/impulsivity

The test for heterogeneity was not significant for any of the hyperactivity/impulsivity measurements, indicating that the variance of SMD was not large enough to be attributed to sampling error only. The between-group analysis of all control groups resulted in a significant small effect size favoring NF (SMD = 0.32; 95% CI 0.15, 0.49) at pre-FU, pre-post was not significant. When including only non-active control groups (done for comparison with inattention, not due to heterogeneity), results favored NF with a significant small effect for the pre-post (SMD = 0.25; 95% CI 0.05, 0.45) measurement and a small effect for pre-FU (SMD = 0.39; 95% CI 0.19, 0.59). Including only active controls resulted in no significant findings for either pre-post or pre-FU. For both control analyses post-FU was not significant; however, post-FU did show a trend toward significance favoring neurofeedback over all control groups (*p* = 0.08; SMD = 0.15; 95% CI − 0.02, 0.32].

### Sensitivity meta-analysis: ‘standard’ NF training

See Tables S-7 and S-8 for details regarding this analysis.

#### Inattention

The tests for heterogeneity as well as effect sizes were similar to those seen in the within-group and between-group analyses including all studies, with slightly stronger effect sizes seen for the sensitivity analysis regarding pre-post and pre-FU time points (increase in SMD ranging from 0.01 to 0.14), however, with small changes in both directions for post-FU (change ranging from − 0.02 decrease to 0.02 increase).

#### Hyperactivity/impulsivity

The tests for heterogeneity as well as effect sizes were similar to those seen in the within-group and between-group analyses including all participants (decrease of 0.01 SMD to increases in SMD up to 0.02).

## Discussion

This meta-analysis investigated the effects of neurofeedback and control conditions directly after treatment and during a follow-up period (2–12 months post-treatment), in which no additional neurofeedback sessions or booster sessions were performed. For neurofeedback, a medium SMD for inattention and hyperactivity/impulsivity were found post-treatment, which changed to a large SMD for inattention and remained a medium SMD for hyperactivity/impulsivity at follow-up (relative to baseline). Non-active control groups yielded a significant small effect size at pre-post that was no longer significant at FU for inattention, and there were no significant effects found for the hyperactivity/impulsivity domain. Active controls had significant large effect sizes for inattention and medium effect sizes for hyperactivity at both pre-post and pre-FU. The between-group analysis was found to significantly favor NF over non-active control groups for both inattention and hyperactivity/impulsivity at pre-post (small effect sizes) and pre-FU (small to medium effect sizes). Active controls were found to be significantly superior regarding inattention at pre-post but no longer at follow-up. In summary, focusing on the pre-treatment to follow-up results, neurofeedback was superior to non-active control groups and similarly effective for inattention and hyperactivity/impulsivity compared to active treatments. These findings provide evidence that there are sustained clinical benefits after neurofeedback and active treatments over an average 6–12 month follow-up period, whereas effects of non-active control groups are no longer significant at FU.

The significant improvement in symptoms at FU for both inattention and hyperactivity–impulsivity in the neurofeedback conditions indicates that NF results in lasting effects for approximately 6 months and potentially up to 1 year. Comparison of effect-sizes between neurofeedback and active control groups showed overlapping confidence intervals (also visualized in Figure S-3), and no significant difference in the between-group analysis, suggesting NF and active controls having similar effects in the respective FU period; however, this finding needs to be viewed with caution due to the small number of studies in the active controls groups (*K* = 4). The tendency in the within-group analyses for a small further improvement in the NF group (inattention: SMD = 0.14; hyperactivity/impulsivity: SMD = 0.11) from post-treatment to FU, albeit not significant, is in line with the further improvement effects seen after two-year follow-up [6, 41]. This tendency was non-existent and moves primarily in the opposite direction (SMD = − 0.1 to 0) for the active and non-active controls (see Figure S-3). Accordingly, post-FU results of the between-group analyses were slightly in favor of NF (SMDs between 0.1 and 0.2, with a statistical trend for hyperactivity/impulsivity) suggesting that effects may become significant with further studies available. In any case, current results do support the sustainability of the clinical benefits of neurofeedback after cessation of treatment.

Regarding the use of medication, we only focused on follow-up periods of 2–12 months, and effects for active treatments demonstrated sustained clinical benefit for these periods (in which children were actively taking medication). This is line with previous studies which demonstrated clinical benefits of psychostimulant medication at 12 months [42] and 24 months [3, 4]; however, the clinical benefits of psychostimulant medication (when naturalistically assessed) are not empirically supported for longer follow-up periods of 2–8 years [43–45]. Despite this, recent epidemiological studies have found that continued controlled medication intake can have positive benefits for patients with ADHD [46–48]. However, Swanson et al. [49] reported that at 12–16-year follow-up of long-term medication use (both consistent and inconsistent use over this time period) was not associated with reduced symptom severity, but it was associated with decreased adult height. These contrasting findings suggest that while medication may have some long-term benefits, the adherence of medication intake may be problematic and long-term medication exposure may be related to potential physical side effects. The follow-up periods used in our study were probably not long enough to demonstrate the decreased efficacy of medication as reported in the above studies [43–45], and since these studies were controlled during the FU periods and not naturalistic, it is likely that medication adherence was high. While NF follow-up treatment effects have not been studied for such long-time intervals, the short-term clinical effects of NF appear to be sustained (without continued training) for an average 2–12 month FU, suggesting potential promise of this approach for sustained clinical benefit in ADHD.

Interestingly, a strong point of our findings is that, despite past heterogeneity in the application of neurofeedback protocols [5], most of the studies included in this paper used ‘standard NF protocols’, the exceptions being Arnold et al. [25] and Bink et al. [26]. Additionally, Arnold et al. [25] used an ‘entertaining’ NF protocol that may not be compatible with principles of learning theory. However, the use of uniform protocols by most of the papers is also evidenced by the absence of significant heterogeneity. Considering only standard protocols, we found that the results supporting NF over non-active controls are slightly strengthened when only including standard NF protocols. This finding supports the continued use of these protocols for future NF studies. Additionally, our significant findings are in line with those of Cortese et al. [8] who demonstrated significant between-group effects even for ‘probably blinded’ ratings (teacher ratings) at post-treatment when only standard NF protocols for total ADHD and inattention symptoms were considered.

When considering placebo effects of NF training, they may still operate at follow-up but we are not aware of tendencies for further improvements of placebo effects for other treatments of other disorders. NF treatment is found to be superior to non-active controls in this analysis, and the effects of non-active controls were not significant at FU, neither for inattention nor hyperactivity/impulsivity. These findings support the idea that NF does indeed have a different, specific effect due to its actual training and not simply due to the non-specific or placebo effects related to the setting, the therapist–patient relationship or expectations. To verify this more RCT’s using controls that closely mimic NF training are required.

## Possible limitations and open questions

Results of the current meta-analysis should be interpreted in line with its limitations.

While some of the studies included here do simultaneously use medication and NF (which may influence the results), the number of participants taking medication did not change for most studies, (only in two studies the NF participants began taking medication between pre- and FU measurement [25, 37]), suggesting that medication changes are not an explanation for the effects found here. Additionally, while dosage was allowed to be changed in five of these studies, two NF groups decreased dosage and two increased dosage while three control groups increased dosage. These changes should not bias the data in favor of the NF, but rather suggest that the NF effects may be slightly masked by the dosage increase seen in the control groups.

When comparing the between-group effect sizes for pre- to post-treatment between this study (Fig. 3) and the latest EAGG meta-analysis by Cortese et al. [8], a small effect for inattention (SMD = 0.36) and hyperactivity/impulsivity (SMD = 0.26) was found. In the current study, SMD’s are lower when including all controls (inattention: SMD = 0.09; hyperactivity/impulsivity: SMD = 0.16) and nearly identical when considering only non-active controls (inattention: SMD = 0.38; hyperactivity/impulsivity SMD = 0.25). This indicates that the studies we have included are representative and not biased towards more effective studies (opposite file-drawer problem, i.e. higher likelihood that positive studies are more often published). But, while we attempted to address the file drawer problem by assessing the fail-safe numbers for our analysis, a potential reporting bias cannot be definitively excluded. However, our finding of much larger fail-safe numbers for NF (generally ≥ 100) than for control conditions (active < 56, non-active < 12) do suggest that the NF condition results are probably not influenced by a reporting bias.

Inherently when investigating FU periods, there are additional limitations involved including completer bias (a bias is introduced because of the factors that cause a person to be involved during a FU time-point) and the lack of an intention to treat analysis (ITT) available for studies using a FU time point. This type of analysis is considered more conservative than the per protocol analyses found in the majority of the papers included here. We chose specifically to not run a risk of bias assessment because this model does not work well for NF studies since it relies heavily on blinding, which poses a problem since most NF studies were not blinded.

As already mentioned, more carefully designed RCTs with longer follow-up time periods are needed before definite conclusions can be drawn. However, the meta-analysis of Cortese et al. [8] on the acute effects of NF was comprised of studies with a comparable design (“well-controlled”) and about the same number of participants (ca. 500). Therefore, this meta-analysis on follow-up effects at the present time may also allow us to derive the first relevant conclusions about the lasting effects of NF treatment.

Future research should focus on addressing both post- and FU-effects of NF and other non-pharmacological treatments for ADHD. Additionally, based on the current findings of within-group effects, placebo or non-specific treatment effects in NF cannot be ruled out and better controls for these effects should continue to be investigated. Finally, it should be noted that the specificity of neurofeedback effects cannot only be derived from RCTs and a meta-analysis of RCTs investigating behavioral outcome. Associations between the behavioral and neurophysiological level (e.g., neuroregulation skills) have already been documented with respect to the post-treatment outcome [17, 50, 51], but are largely missing from the literature that has conducted FU measurements. These parameters provide an additional method to evaluate treatment effects and should be included in future research of long-term follow-ups.

## Conclusion

Our meta-analytic results of NF treatment follow-up suggest that there are sustained symptom reductions over time in comparison with non-active control conditions. The improvements seen here are comparable to active treatments (including methylphenidate) at a short-term FU of 2–12 months. As such, NF can be considered a non-pharmacological treatment option for ADHD with evidence of treatment effects that are sustained when treatment is completed and withdrawn. Future research should focus on the comparison of standardized NF treatments with standardized control treatments, controlling for unspecific effects and changes in additional treatments (medication). Given the need for additional treatments for ADHD with long-term outcomes, clinical trials of NF should aim for primary outcome measures that compare pre-treatment with systematic long-term follow-up behavioral ratings, to address sustainability of effects.

## Electronic supplementary material

Below is the link to the electronic supplementary material.

Supplementary material 1 (DOCX 458 kb)
