# Supplementary material for: Sustained effects of neurofeedback in ADHD: a systematic review and meta-analysis
Source: Eur Child Adolesc Psychiatry. 2018 Feb 14;28(3):293–305. doi: 10.1007/s00787-018-1121-4 (PMC6404655; doi:10.1007/s00787-018-1121-4)
Supplement: Supplementary file 1 — Supplementary material 1 (DOCX 458 kb) [file 787_2018_1121_MOESM1_ESM.docx]

**Supplementary Material**

Sustained effects of Neurofeedback in ADHD: A systematic review and meta-analysis

Jessica Van Doren, Martijn Arns, Hartmut Heinrich, Madelon A. Vollebregt, Ute Strehl, Sandra K. Loo

| Contents | Page # |
| --- | --- |
| Figure S-1: PRISMA Flow Diagram | 2 |
| Table S-1: Prisma Checklist | 3 |
| Figure S-2: Explanation of Standard NF | 6 |
| Table S-2: Control Conditions For NF | 7 |
| Table S-3: Excluded Studies, with reasons | 8 |
| Table S-4: Included Studies: additional information | 14 |
| Table S-5: NF group data: extracted data and RevMan effect sizes | 18 |
| Table S-6: Control group data: extracted data and RevMan effect sizes | 19 |
| Figure S-3: Bar Graph within-group Analysis | 20 |
|  |  |
| Sensitivity Standard NF Meta-Analysis |  |
| Table S-7: Senstitivity analysis for within- and between-group values for the inattention domain. | 21 |
| Table S-8: Senstitivity analysis for within- and between-group values for the hyperactivity/impulsivity domain. | 22 |

Figure S-1: Preferred Reporting Items for Systematic Reviews and Meta-Analyses (PRISMA) flow diagram of study selection (final search: 29^th^ of November 2017). See supplement for list of excluded articles with reason for exclusion.

Studies included in quantitative synthesis (meta-analysis)
(n = 10)

Records excluded
(n = 647)

Records screened
(n = 723)

Records after duplicates removed
(n = 723)

## Identification

## Eligibility

## Included

## Screening

Additional records identified through other sources
(n = 4)

Records identified through database searching
(n = 3657)

Full-text articles assessed for eligibility
(n = 76)

Full-text articles excluded, not meeting inclusion criteria (n=66)
(n = 56)

Studies included in qualitative synthesis
(n = 10)

**Table S-1: PRISMA Checklist**

| **Section/topic** | **#** | **Checklist item** | **Reported on page #** |
| --- | --- | --- | --- |
| **TITLE** | | |  |
| Title | 1 | Identify the report as a systematic review, meta-analysis, or both. | 1 |
| **ABSTRACT** | | |  |
| Structured summary | 2 | Provide a structured summary including, as applicable: background; objectives; data sources; study eligibility criteria, participants, and interventions; study appraisal and synthesis methods; results; limitations; conclusions and implications of key findings; systematic review registration number. | 2 |
| **INTRODUCTION** | | |  |
| Rationale | 3 | Describe the rationale for the review in the context of what is already known. | 3-5 |
| Objectives | 4 | Provide an explicit statement of questions being addressed with reference to participants, interventions, comparisons, outcomes, and study design (PICOS). | 5 |
| **METHODS** | | |  |
| Protocol and registration | 5 | Indicate if a review protocol exists, if and where it can be accessed (e.g., Web address), and, if available, provide registration information including registration number. | 5 |
| Eligibility criteria | 6 | Specify study characteristics (e.g., PICOS, length of follow-up) and report characteristics (e.g., years considered, language, publication status) used as criteria for eligibility, giving rationale. | 6 |
| Information sources | 7 | Describe all information sources (e.g., databases with dates of coverage, contact with study authors to identify additional studies) in the search and date last searched. | 5 |
| Search | 8 | Present full electronic search strategy for at least one database, including any limits used, such that it could be repeated. | 5 |
| Study selection | 9 | State the process for selecting studies (i.e., screening, eligibility, included in systematic review, and, if applicable, included in the meta-analysis). | 5-6 |
| Data collection process | 10 | Describe method of data extraction from reports (e.g., piloted forms, independently, in duplicate) and any processes for obtaining and confirming data from investigators. | 6-7 |
| Data items | 11 | List and define all variables for which data were sought (e.g., PICOS, funding sources) and any assumptions and simplifications made. | 6-7 |
| Risk of bias in individual studies | 12 | Describe methods used for assessing risk of bias of individual studies (including specification of whether this was done at the study or outcome level), and how this information is to be used in any data synthesis. | No individual risk of bias performed |
| Summary measures | 13 | State the principal summary measures (e.g., risk ratio, difference in means). | 6-8 |
| Synthesis of results | 14 | Describe the methods of handling data and combining results of studies, if done, including measures of consistency (e.g., I^2^) for each meta-analysis. | 7-8 |
| Risk of bias across studies | 15 | Specify any assessment of risk of bias that may affect the cumulative evidence (e.g., publication bias, selective reporting within studies). | No risk of bias assessed |
| Additional analyses | 16 | Describe methods of additional analyses (e.g., sensitivity or subgroup analyses, meta-regression), if done, indicating which were pre-specified. | 7-8 |
| **RESULTS** | | |  |
| Study selection | 17 | Give numbers of studies screened, assessed for eligibility, and included in the review, with reasons for exclusions at each stage, ideally with a flow diagram. | 8-9; Tables S-3 and S-4 |
| Study characteristics | 18 | For each study, present characteristics for which data were extracted (e.g., study size, PICOS, follow-up period) and provide the citations. | Table 1 |
| Risk of bias within studies | 19 | Present data on risk of bias of each study and, if available, any outcome level assessment (see item 12). | Not performed |
| Results of individual studies | 20 | For all outcomes considered (benefits or harms), present, for each study: (a) simple summary data for each intervention group (b) effect estimates and confidence intervals, ideally with a forest plot. | Figure 1 to 3 |
| Synthesis of results | 21 | Present results of each meta-analysis done, including confidence intervals and measures of consistency. | 9-11; Tables S-5 and S-6 |
| Risk of bias across studies | 22 | Present results of any assessment of risk of bias across studies (see Item 15). | Not conducted |
| Additional analysis | 23 | Give results of additional analyses, if done (e.g., sensitivity or subgroup analyses, meta-regression [see Item 16]). | 10 - 11; Tables S-7 and S-8 |
| **DISCUSSION** | | |  |
| Summary of evidence | 24 | Summarize the main findings including the strength of evidence for each main outcome; consider their relevance to key groups (e.g., healthcare providers, users, and policy makers). | 12-14 |
| Limitations | 25 | Discuss limitations at study and outcome level (e.g., risk of bias), and at review-level (e.g., incomplete retrieval of identified research, reporting bias). | 14-15 |
| Conclusions | 26 | Provide a general interpretation of the results in the context of other evidence, and implications for future research. | 15- 16 |
| **FUNDING** | | |  |
| Funding | 27 | Describe sources of funding for the systematic review and other support (e.g., supply of data); role of funders for the systematic review. | 16 |

*From:*  Moher D, Liberati A, Tetzlaff J, Altman DG, The PRISMA Group (2009). Preferred Reporting Items for Systematic Reviews and Meta-Analyses: The PRISMA Statement. PLoS Med 6(7): e1000097. doi:10.1371/journal.pmed1000097

**Figure S-2:** Explanation of Standard NF protocols as a function of training site. Theta/Beta neurofeedback protocols are specfically trained at Fz or Cz, in line with most studies that have assessed the Theta/Beta ratio at those sites; SCP neurofeedback is always applied at Cz and SMR neurofeedback is always applied at a site that overlies the sensori-motor strip, i.e. C3, Cz or C4. Theta/Beta neurofeedback is focused on downtraining Theta and rewarding Beta, SMR is focused on SMR enhancement and SCP neurofeedback is focused on bidirectional training of negativation and positivation.

**
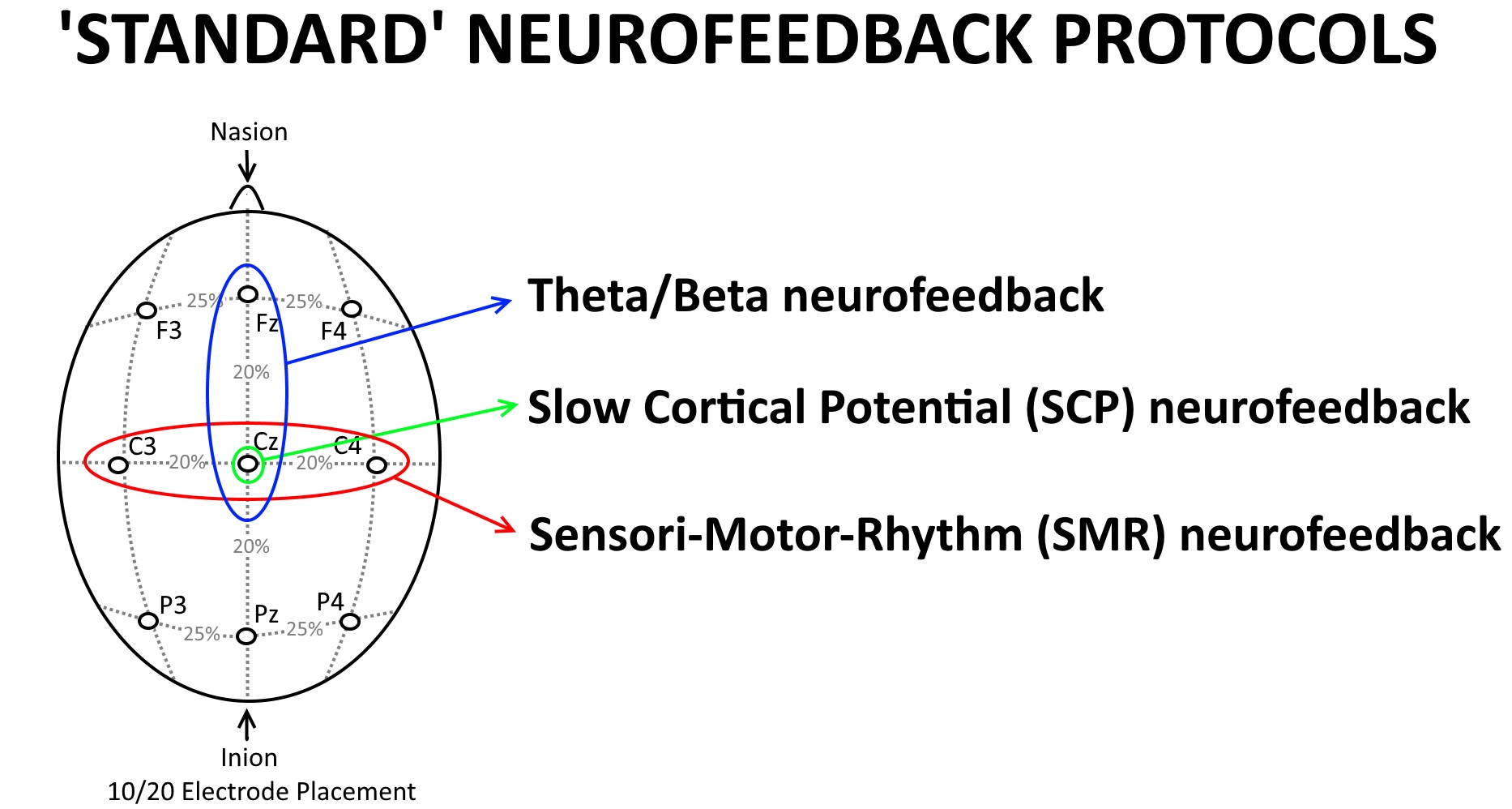
**

**Table S-2:** Control conditions for neurofeedback

| Type of Control Group | Explanation | Forms of Control Group |
| --- | --- | --- |
| Active Control | Control conditions that have been clinically proven to be effective treatments for ADHD | Medication (e.g., methylphenidate)  Self-management training |
| Non-active Control | All control conditions that have not been proven to provide specific clinical benefit in the treatment of ADHD. Includes inactive, semi-active controls and placebo controls. | Waiting list controls  Attention training  Cognitive training  Treatment as usual  Sham control |
| *Inactive Control | Control conditions in which the children do nothing | Waiting list controls |
| *Semi-active controls | Control conditions in which the children participate in a type of therapy in order to control for non-specific effects of training. However these methods have not yet been proven and accepted as effective treatments for ADHD. | Attention training  Cognitive training  Treatment as usual |
| *Placebo Control | Control condition in which children must participate in a neurofeedback training, in which for example a yoked control is used, for example by playback of a prerecorded EEG of another participant instead of their own EEG activity. | Sham neurofeedback |

*For our analysis purposes, inactive, semi-active and placebo control conditions were grouped together to be compared to active control conditions. These are referred to as 'non-active'.

| **Table S-3:** Excluded studies, with reasons (alphabetical order). |  |
| --- | --- |
| **Excluded Articles** | **Reasons for exclusion** |
| Alirezaloo N, Hosseini AS, Pishyareh E, Nosratabadi M, Haghgoo HA, Biglarian A. Investigation of the impact of QEEG-based biofeedback on attention and behavioral features in young male adolescents with ADHD. Journal of Advanced Medical Sciences and Applied Technologies. 2016;2(2):204-212. | Not randomized (matched based on qEEG profiles) |
| Albrecht JS, Bubenzer-Busch S, Gallien A, Knospe EL, Gaber TJ, Zepf FD. Effects of a structured 20-session slow-cortical-potential-based neurofeedback program on attentional performance in children and adolescents with attention-deficit hyperactivity disorder: retrospective analysis of an open-label pilot-approach and 6-month follow-up. Neuropsychiatr Dis Treat. 2017;13:667-683. | Not randomized (no comparison group) |
| Bakhshayesh AR, Hansch S, Wyschkon A, Rezai MJ, Esser G. Neurofeedback in ADHD: a single-blind randomized controlled trial. Eur Child Adolesc Psychiatry. 2011;20(9):481-491. | Only pre-post data reported in the paper. No response from authors regarding further data. |
| Bakhtadze S, Beridze M, Geladze N, Khachapuridze N, Bornstein N. Effect of EEG biofeedback on cognitive flexibility in children with attention deficit hyperactivity disorder with and without epilepsy. Appl Psychophysiol Biofeedback. 2016;41(1):71-79. | Only pre-post data reported in the paper. No response from authors regarding further data. |
| Baumeister S, Wolf I, Holz N, Boecker-Schlier R, Adamo N, Holtmann M, et al. Neurofeedback training effects on inhibitory brain activation in ADHD: A matter of learning? Neuroscience. 2016. | Insufficient FU data (5 participants total) |
| Beauregard M, Levesque J. Functional magnetic resonance imaging investigation of the effects of neurofeedback training on the neural bases of selective attention and response inhibition in children with attention-deficit/hyperactivity disorder. Appl Psychophysiol Biofeedback. 2006;31(1):3-20. | No FU data |
| Bink M, van Nieuwenhuizen C, Popma A, Bongers IL, van Boxtel GJ. Neurocognitive effects of neurofeedback in adolescents with ADHD: a randomized controlled trial. J Clin Psychiatry. 2014;75(5):535-542. | No FU data (FU paper included in current meta-analysis) |
| Bink M, van Nieuwenhuizen C, Popma A, Bongers IL, van Boxtel GJ. Behavioral effects of neurofeedback in adolescents with ADHD: a randomized controlled trial. Eur Child Adolesc Psychiatry. 2015;24(9):1035-1048. | No FU data (FU paper included in current meta-analysis) |
| Bluschke A, Broschwitz F, Kohl S, Roessner V, Beste C. The neuronal mechanisms underlying improvement of impulsivity in ADHD by theta/beta neurofeedback. Sci Rep. 2016;6:31178. | No randomization |
| Cowley B, Holmstrom E, Juurmaa K, Kovarskis L, Krause CM. Computer enabled neuroplasticity treatment: A clinical trial of a novel design for neurofeedback therapy in adult ADHD. Front Hum Neurosci. 2016;10:205. | Adults |
| DeBeus RJ, Kaiser DA. Neurofeedback with children with attention deficit hyperactivity disorder. A randomized double-blind placebo-controlled study. Neurofeedback and Neuromodulation Techniques and Applications 2011. p. 127-152. | No FU data |
| Deilami M, Jahandideh A, Kazemnejad Y, Fakour Y, Alipoor S, Rabiee F, et al. The effect of neurofeedback therapy on reducing symptoms associated with attention deficit hyperactivity disorder: A case series study. Basic Clin Neurosci. 2016;7(2):167-171. | Case study |
| Dopfner M, Hautmann C, Dose C, Banaschewski T, Becker K, Brandeis D, et al. ESCAschool study: trial protocol of an adaptive treatment approach for school-age children with ADHD including two randomised trials. BMC Psychiatry. 2017;17(1):269. | Still recruiting participants |
| Drechsler R, Straub M, Doehnert M, Heinrich H, Steinhausen HC, Brandeis D. Controlled evaluation of a neurofeedback training of slow cortical potentials in children with attention deficit/hyperactivity disorder (ADHD). Behav Brain Funct. 2007;3:35. | Incomplete randomization |
| Flisiak-Antonijczuk H, Adamowska S, Chladziñska-Kiejna S, Kalinowski R, Adamowski T. Treatment of ADHD: Comparison of EEG-biofeedback and methylphenidate. Archives of Psychiatry and Psychotherapy. 2015;17(4):32-38. | Not randomized |
| Fuchs T, Birbaumer N, Lutzenberger W, Gruzelier JH, Kaiser J. Neurofeedback treatment for attention-deficit/hyperactivity disorder in children: a comparison with methylphenidate. Appl Psychophysiol Biofeedback. 2003;28(1):1-12. | Not randomized |
| Gani C, Birbaumer N, Strehl U. Long term effects after feedback of slow cortical potentials and of theta-beta-amplitudes in children with attention deficit/hyperactivity disorder (ADHD). Int J Bioelectromagn. 2008;10(4):209-232. | 22 month FU |
| Gelade K, Bink M, Janssen TW, van Mourik R, Maras A, Oosterlaan J. An RCT into the effects of neurofeedback on neurocognitive functioning compared to stimulant medication and physical activity in children with ADHD. Eur Child Adolesc Psychiatry. 2016. | No FU data (FU paper included in current meta-analysis) |
| Gelade K, Janssen TW, Bink M, van Mourik R, Maras A, Oosterlaan J. Behavioral effects of neurofeedback compared to stimulants and physical activity in attention-deficit/hyperactivity disorder: A randomized controlled trial. J Clin Psychiatry. 2016;77(10):e1270-e1277. | No FU data (FU paper included in current meta-analysis) |
| Gevensleben H, Holl B, Albrecht B, Schlamp D, Kratz O, Studer P, et al. Distinct EEG effects related to neurofeedback training in children with ADHD: a randomized controlled trial. Int J Psychophysiol. 2009;74(2):149-157. | No FU data (FU paper included in current meta-analysis) |
| Gevensleben H, Holl B, Albrecht B, Vogel C, Schlamp D, Kratz O, et al. Is neurofeedback an efficacious treatment for ADHD? A randomised controlled clinical trial. J Child Psychol Psychiatry. 2009;50(7):780-789. | No FU data (FU paper included in current meta-analysis) |
| Gevensleben H, Kleemeyer M, Rothenberger LG, Studer P, Flaig-Rohr A, Moll GH, et al. Neurofeedback in ADHD: further pieces of the puzzle. Brain Topogr. 2014;27(1):20-32. | Study 1: not randomized  Study 2: no primary diagnosis of ADHD required |
| Gonzalez-Castro P, Cueli M, Rodriguez C, Garcia T, Alvarez L. Efficacy of neurofeedback versus pharmacological support in subjects with ADHD. Appl Psychophysiol Biofeedback. 2016;41(1):17-25. | No randomization |
| Habibollahi S, Souri A, Arbabi FH, Ashoori J. Effects of neurofeedback training on sustain attention and planning in students with attention deficit disorder. Koomesh. 2016;17(2):447-454. | Non-English manuscript |
| Holtmann M, Grasmann D, Cionek-Szpak E, Hager V, Panzner N, Beyer A, et al. Specific effects of neurofeedback on impulsivity in ADHD. Kindheit und Entwicklung. 2009;18(2):95-104. | No FU data |
| Janssen TW, Bink M, Gelade K, van Mourik R, Maras A, Oosterlaan J. A randomized controlled trial investigating the effects of neurofeedback, methylphenidate, and physical activity on event-related potentials in children with attention-deficit/hyperactivity disorder. J Child Adolesc Psychopharmacol. 2016;26(4):344-353. | No FU data (FU paper included in current meta-analysis) |
| Janssen TW, Bink M, Gelade K, van Mourik R, Maras A, Oosterlaan J. A randomized controlled trial into the effects of neurofeedback, methylphenidate, and physical activity on EEG power spectra in children with ADHD. J Child Psychol Psychiatry. 2016;57(5):633-644. | No FU data (FU paper included in current meta-analysis) |
| Kaiser DA, Othmer S. Effect of neurofeedback on variables of attention in a large multi-center trial. Journal of Neurotherapy. 2000;4(1):5-15. | Not randomized |
| Kim SK, Yoo EY, Lee JS, Jung MY, Park SH, Park JH. The effects of neurofeedback training on concentration in children with attention deficit / Hyperactivity disorder. International Journal of Bio-Science and Bio-Technology. 2014;6(4):13-24. | Not randomized |
| Kropotov JD, Grin-Yatsenko VA, Ponomarev VA, Chutko LS, Yakovenko EA, Nikishena IS. ERPs correlates of EEG relative beta training in ADHD children. Int J Psychophysiol. 2005;55(1):23-34. | Not randomized |
| Lansbergen MM, van Dongen-Boomsma M, Buitelaar JK, Slaats-Willemse D. ADHD and EEG-neurofeedback: a double-blind randomized placebo-controlled feasibility study. J Neural Transm (Vienna). 2011;118(2):275-284. | No FU data |
| Lee N, Park S, Kim J. Hippotherapy and neurofeedback training effect on the brain function and serum brain-derived neurotrophic factor level changes in children with attention-deficit or/and hyperactivity disorder. J Exerc Nutrition Biochem. 2017;21(3):35-42. | Only pre-post data reported in the paper. No response from authors regarding further data. |
| Lee EJ, Jung CH. Additive effects of neurofeedback on the treatment of ADHD: A randomized controlled study. Asian Journal of Psychiatry. 2017;25:16-21. | No response |
| Leins U, Goth G, Hinterberger T, Klinger C, Rumpf N, Strehl U. Neurofeedback for children with ADHD: a comparison of SCP and Theta/Beta protocols. Appl Psychophysiol Biofeedback. 2007;32(2):73-88. | Randomized between neurofeedback groups |
| Levesque J, Beauregard M, Mensour B. Effect of neurofeedback training on the neural substrates of selective attention in children with attention-deficit/hyperactivity disorder: a functional magnetic resonance imaging study. Neurosci Lett. 2006;394(3):216-221. | Only pre-post data reported in the paper. No response from authors regarding further data. |
| Linden M, Habib T, Radojevic V. A controlled study of the effects of EEG biofeedback on cognition and behavior of children with attention deficit disorder and learning disabilities. Biofeedback Self Regul. 1996;21(1):35-49. | Only pre-post data reported in the paper. No response from authors regarding further data. |
| Liu T, Wang J, Chen Y, Wang R, Song M. Neurofeedback treatment experimental study for ADHD by using the brain-computer interface neurofeedback system. IFMBE Proceedings; 2013. p. 1537-1540. | Only pre-post data reported in the paper. No response from authors regarding further data. |
| Lubar JF, Swartwood MO, Swartwood JN, O'Donnell PH. Evaluation of the effectiveness of EEG neurofeedback training for ADHD in a clinical setting as measured by changes in T.O.V.A. scores, behavioral ratings, and WISC-R performance. Biofeedback Self Regul. 1995;20(1):83-99. | Not randomized |
| Marx AM, Ehlis AC, Furdea A, Holtmann M, Banaschewski T, Brandeis D, et al. Near-infrared spectroscopy (NIRS) neurofeedback as a treatment for children with attention deficit hyperactivity disorder (ADHD)-a pilot study. Front Hum Neurosci. 2014;8:1038. | Unable to share NF clinical data prior to publication |
| Maurizio S, Liechti MD, Heinrich H, Jancke L, Steinhausen HC, Walitza S, et al. Comparing tomographic EEG neurofeedback and EMG biofeedback in children with attention-deficit/hyperactivity disorder. Biol Psychol. 2014;95:31-44. | No FU data |
| Mohammadi MR, Malmir N, Khaleghi A. Comparison of sensorimotor rhythm (SMR) and beta training on selective attention and symptoms in children with attention deficit/hyperactivity disorder (ADHD): A trend report. Iran J Psychiatry. 2015;10(3):165-174. | No control group |
| Mohagheghi A, Amiri S, Moghaddasi Bonab N, Chalabianloo G, Noorazar SG, Tabatabaei SM, et al. A Randomized Trial of Comparing the Efficacy of Two Neurofeedback Protocols for Treatment of Clinical and Cognitive Symptoms of ADHD: Theta Suppression/Beta Enhancement and Theta Suppression/Alpha Enhancement. Biomed Res Int. 2017;2017:3513281. | Randomized between neurofeedback groups |
| Mohtasham M, Eftekharsaadi Z. Determination of the effectiveness of neurofeedback on reducing the symptoms of hyperactivity and increasing the accuracy and caution in ADHD children. Asian Social Science. 2016;12(10):222-227. | Not randomized |
| Monastra VJ, Monastra DM, George S. The effects of stimulant therapy, EEG biofeedback, and parenting style on the primary symptoms of attention-deficit/hyperactivity disorder. Appl Psychophysiol Biofeedback. 2002;27(4):231-249. | Not randomized |
| Moreno-García I, Delgado-Pardo G, Camacho-Vara de Rey C, Meneres-Sancho S, Servera-Barceló M. Neurofeedback, pharmacological treatment and behavioral therapy in hyperactivity: Multilevel analysis of treatment effects on electroencephalography. International Journal of Clinical and Health Psychology. 2015;15(3):217-225. | No FU data |
| Nazari M, Querne L, Broca A, Berquin P. Effectiveness of EEG biofeedback as compared with methylphenidate in the treatment of attention-deficit/hyperactivity disorder: A clinical out-come study. Neuroscience and Medicine. 2011;2(2):78-86. | Not randomized |
| Nooner KB, Leaberry KD, Keith JR, Ogle RL. Clinic outcome assessment of a brief course neurofeedback for childhood ADHD Symptoms. J Behav Health Serv Res. 2016. | Not randomized |
| Ogrim G, Hestad KA. Effects of neurofeedback versus stimulant medication in attention-deficit/hyperactivity disorder: a randomized pilot study. J Child Adolesc Psychopharmacol. 2013;23(7):448-457. | No FU data |
| Okumura Y, Kita Y, Omori M, Suzuki K, Yasumura A, Fukuda A, et al. Predictive factors of success in neurofeedback training for children with ADHD. Dev Neurorehabil. 2017:1-10. | not randomized |
| Pahlevanian A, Alirezaloo N, Naghel S, Alidadi F, Nejati V, Kianbakht M. Neurofeedback associated with neurocognitive-rehabilitation training on children with attention-deficit/hyperactivity disorder (ADHD). International Journal of Mental Health and Addiction. 2015:1-10. | Only pre-post data reported in the paper. No response from authors regarding further data. |
| Perreau-Linck E, Lessard N, Lévesque J, Beauregard M. Effects of neurofeedback training on inhibitory capacities in ADHD children: a single-blind, randomized, placebo-controlled study. J Neurotherapy. 2010;14:229-42 | Insufficient sample size (5 NF, 4 Control) |
| Rossiter T. The effectiveness of neurofeedback and stimulant drugs in treating AD/HD: part II. Replication. Appl Psychophysiol Biofeedback. 2004;29(4):233-243. | Not randomized |
| Rossiter TR, La Vaque TJ. A comparison of eeg biofeedback and psychostimulants in treating attention deficit/hyperactivity disorders. Journal of Neurotherapy. 1995;1(1):48-59. | Not randomized |
| Russell-Chapin L, Kemmerly T, Liu WC, Zagardo MT, Chapin T, Dailey D, et al. The effects of neurofeedback in the default mode network: Pilot study results of medicated children with ADHD. Journal of Neurotherapy. 2013;17(1):35-42. | No clinical data, no FU |
| Shin MS, Jeon H, Kim M, Hwang T, Oh SJ, Hwangbo M, et al. Effects of Smart-Tablet-Based Neurofeedback Training on Cognitive Function in Children with Attention Problems. J Child Neurol. 2016;31(6):750-760. | 1 month FU |
| Steiner NJ, Frenette EC, Rene KM, Brennan RT, Perrin EC. Neurofeedback and cognitive attention training for children with attention-deficit hyperactivity disorder in schools. J Dev Behav Pediatr. 2014;35(1):18-27. | No FU data |
| Steiner NJ, Sheldrick RC, Gotthelf D, Perrin EC. Computer-based attention training in the schools for children with attention deficit/hyperactivity disorder: a preliminary trial. Clin Pediatr (Phila). 2011;50(7):615-622. | No FU data |
| Strehl U, Aggensteiner P, Wachtlin D, Brandeis D, Albrecht B, Arana M, et al. Neurofeedback of Slow Cortical Potentials in Children with Attention-Deficit/Hyperactivity Disorder: A Multicenter Randomized Trial Controlling for Unspecific Effects. Front Hum Neurosci. 2017;11:135. | Cannot share FU data before publication |
| Strehl U, Leins U, Goth G, Klinger C, Hinterberger T, Birbaumer N. Self-regulation of slow cortical potentials: a new treatment for children with attention-deficit/hyperactivity disorder. Pediatrics. 2006;118(5):e1530-1540. | Randomized between neurofeedback groups |
| Takahashi J, Yasumura A, Nakagawa E, Inagaki M. Changes in negative and positive EEG shifts during slow cortical potential training in children with attention-deficit/hyperactivity disorder: a preliminary investigation. Neuroreport. 2014;25(8):618-624. | Not randomized |
| Thomas BL, Viljoen M. EEG brain wave activity at rest and during evoked attention in children with attention-deficit/hyperactivity disorder and effects of methylphenidate. Neuropsychobiology. 2016;73(1):16-22. | No symptom or behavioral data |
| van Dongen-Boomsma M, Vollebregt MA, Slaats-Willemse D, Buitelaar JK. A randomized placebo-controlled trial of electroencephalographic (EEG) neurofeedback in children with attention-deficit/hyperactivity disorder. J Clin Psychiatry. 2013;74(8):821-827. | No FU data to share due to large number of participants beginning or changing medication between post and FU |
| Vollebregt MA, van Dongen-Boomsma M, Buitelaar JK, Slaats-Willemse D. Does EEG-neurofeedback improve neurocognitive functioning in children with attention-deficit/hyperactivity disorder? A systematic review and a double-blind placebo-controlled study. J Child Psychol Psychiatry. 2014;55(5):460-472. | No FU data to share due to large number of participants beginning or changing medication between post and FU |
| Wang Z. Neurofeedback training intervention for enhancing working memory function in attention deficit and hyperactivity disorder (ADHD) Chinese students. NeuroQuantology. 2017;15(2):277-283. | Only pre-post data reported in the paper. No response from authors regarding further data. |
| Wangler S, Gevensleben H, Albrecht B, Studer P, Rothenberger A, Moll GH, et al. Neurofeedback in children with ADHD: specific event-related potential findings of a randomized controlled trial. Clin Neurophysiol. 2011;122(5):942-950. | No FU data (FU paper included in current meta-analysis) |
| Xiong Z, Shi S, Xu H. A controlled study of the effectiveness of EEG biofeedback training on-children with attention deficit hyperactivity disorder. J Huazhong Univ Sci Technolog Med Sci. 2005;25(3):368-370. | Not randomized |

**Table S-4:** Additional information on included studies

| **Included studies** | **Notes** |
| --- | --- |
| Heinrich H, Gevensleben H, Freisleder FJ, Moll GH, Rothenberger A. Training of slow cortical potentials in attention-deficit/hyperactivity disorder: evidence for positive behavioral and neurophysiological effects. Biol Psychiatry. 2004;55(7):772-775. | - Country: Germany   Funding source: This study was supported in part by Hogrefe Verlag,Göttingen and several International Service Clubs from the Göttingen area.   - Trial registration: none - Commentary on study:   - FU data provided by author - Medicated: 6/13 NF (at all time points)   - No medication dose change allowed - ADHD subtypes:   - Combined: 9 NF   - Inattentive: 4 NF |
| Gevensleben H, Holl B, Albrecht B, Schlamp D, Kratz O, Studer P, Rothenberger A, Moll GH, Heinrich H (2010) Neurofeedback training in children with ADHD: 6-month follow-up o﻿f a randomised controlled trial. Eur Child Adolesc Psychiatry 19(9):715–724. https://doi.org/10.1007/s00787-010-0109-5 | - Country: Germany - Funding source: German Research Foundation (DFG) - Trial registration: ISRCTN87071503 - Medicated: 0/38 NF; 0/23 Control (at all time points)   - No medication dose change allowed - Commentary on study:   - 33 dropouts, but dropout percentages did not differ between groups - ADHD subtypes:   - Combined: 23 NF, 17 Control   - Inattentive: 15 NF, 6 Control |
| Duric NS, Assmus J, Gundersen D, Elgen IB. Neurofeedback for the treatment of children and adolescents with ADHD: a randomized and controlled clinical trial using parental reports. BMC Psychiatry. 2012;12:107. | - Country: Norway - Funding source: not reported - Trial registration: NCT01252446 - Medicated: 0/24 NF; 29/29 Control (at all time points)   - No medication dose change allowed - Commentary on study: none - Hyperactivity reported - ADHD subtypes: Not reported |
| Meisel V, Servera M, Garcia-Banda G, Cardo E, Moreno I. Neurofeedback and standard pharmacological intervention in ADHD: a randomized controlled trial with six-month follow-up. Biol Psychol. 2013;94(1):12-21. | - Country: Spain - Funding source: Spanish State Department of Science and Innovation (Plan Nacional I+D+I/PSI2008-06008-C02-01), the Government of Illes Balears and the Social European Fund. (FPI10X-6594122-E). - Trial registration: none - Medicated: 11/11 Control (at all time points), 0/12 NF at pre and post measurement, 2/12 NF at 2 month FU   - No medication dose change allowed - Commentary on study: none - ADHD subtypes:   - Combined: 9 NF, 9 Control - Inattentive: 3 NF, 2 Control |
| Arnold LE, Lofthouse N, Hersch S, Pan X, Hurt E, Bates B, et al. EEG neurofeedback for ADHD: double-blind sham-controlled randomized pilot feasibility trial. J Atten Disord. 2013;17(5):410-419. | - Country: USA - Funding source: National Institute of Mental Health Award R34 MH080775 and award UL1RR025755 from the National Center for Research Resources. - Free use of Smartbrain Technologies supplied by Cyberlearning Technology, LLC. - Trial Registration: none - Medicated: 0 at pre-measurement, 1/25 NF participants at post measurement, 7/25 NF participants at FU measurement. 0/11 Control at all time points.   - Medication dose not reported. - Commentary on study:   - Age and standard deviation of participants reported for entire group (26 NF, 13 Control), but analysis included 25 NF and 11 Control. - ADHD subtypes:   - Combined: 17 NF, 9 Control - Inattentive: 9 NF, 4 Control |
| Li L, Yang L, Zhuo CJ, Wang YF. A randomised controlled trial of combined EEG feedback and methylphenidate therapy for the treatment of ADHD. Swiss Med Wkly. 2013;143:w13838. | - Country: China - Funding source: National Climb Program (95-special-09). Dr. Li Yang received research grant from Janssen Science Council of China. - Trial registration: none - Medicated: 32/32 NF; 32/32 Control (at all time points)   - Medication dose dropped in NF group (Baseline: 19.6 ± 9.8 mg; FU: 15.2 ± 8.2 mg). Control group increased (baseline: 18.6± 7.3 mg; FU: 19.2 ± 7.3 mg) - Commentary on study: none - ADHD subtypes:   - Combined: 9 NF, 10 Control   - Inattentive: 21 NF, 21 Control - Hyperactive/impulsive: 2 NF, 1 Control |
| Steiner NJ, Frenette EC, Rene KM, Brennan RT, Perrin EC. In-school neurofeedback training for ADHD: sustained improvements from a randomized control trial. Pediatrics. 2014;133(3):483-492. | - Country: USA - Funding source: Institute of Education Sciences grant (R305A090100). - Trial registration: NCT01583829 - Medicated: 15/34 NF; 14/34 cognitive training; 20/36 Wait list (at all time points)   - Medication dose: NF maintained with 0.7 mg increase(n.s) while both Control groups significantly increased dose (Cognitive training: 13.08 mg increase; Wait list Control: 9.14 mg increase) - Commentary on study: none - ADHD subtypes: Not reported |
| Christiansen H, Reh V, Schmidt MH, Rief W. Slow cortical potential neurofeedback and self-management training in outpatient care for children with ADHD: study protocol and first preliminary results of a randomized controlled trial. Front Hum Neurosci. 2014;8:943. | - Country: Germany - Funding source: not reported - Trial registration: NCT01879644 - Medicated:   - 1/18 NF at pre and post, 0/18 at FU; 6/21 Control (at all time points)   - Medication dose at FU: Control group: 3 participants increased medication dose, 2 participants maintained dose, 1 participant had no FU data. - Commentary on study   - Follow-up data from this study provided by author - ADHD subtypes:   - Combined: 9 NF, 13 Control   - Hyperactive: 2 NF, 1 Control   - Inattentive: 6 NF, 6 Control - Missing: 1 NF, 1 Control |
| Bink M, Bongers IL, Popma A, Janssen TW, van Nieuwenhuizen C. 1-year follow-up of neurofeedback treatment in adolescents with attention-deficit hyperactivity disorder: randomised controlled trial. BJPsych open. 2016;2(2):107-115. | - Country: The Netherlands - Funding source: The Netherlands Organization for Health Research and Development (ZonMw): 157 002 004. The Netherlands Organization for   Health Research and Development (ZonMw): 157 002 004.   - Trial registration number: Dutch trial register (Ref. no: 1759) - Medication:   - Pre: 19/41 NF; 12/19 Control   - Post: 17/41 NF; 12/19 Control   - FU: 19/41 NF; 11/19 Control   - Medication dose not reported separated by group. - Commentary on study: none - ADHD subtypes: Not reported |
| Gelade K, Janssen TWP, Bink M, Twisk JWR, van Mourik R, Maras A, et al. A 6-month follow-up of an RCT on behavioral and neurocognitive effects of neurofeedback in children with ADHD. Eur Child Adolesc Psychiatry. 2017. | - Country: The Netherlands - Funding source: Netherlands Organization   for Health Research and Development (ZonMw): 157 003 012   - Trial registration number: NCT01363544 - Medicated:   - 0/20 NF; 21/21 MPH Control; 0/17 Exercise Control (at all time points as reported in supplementary analysis)   - Medication dose at FU: dose not reported. Participants in the NF and exercise group did not take medication from pre to post, while all of MPH took medication from pre to post. Changes occurred between post and FU measurements. - Commentary on study: Values from the supplementary analysis were used instead of primary analysis to account for drop-outs and medication change from post to FU measurement. - ADHD subtypes: not reported |

**Table S-5:** NF group data for inattention and hyperactivity/impulsivity domain: extracted data (M ± SD) and RevMan effect sizes
(SMD [95% confidence intervals]).

| Author | Year | Pre | Post | FU | PrePost | PreFU | PostFU |
| --- | --- | --- | --- | --- | --- | --- | --- |
| Inattention | | | | | | | |
| Heinrich et al. | 2004 | 2.03 ± 0.46 | 1.59 ± 0.59 | 1.33 ± 0.48 | 0.81 [0.00, 1.61] | 1.44 [0.56, 2.32] | 0.47 [-0.31, 1.25] |
| Gevensleben et al. | 2010 | 2.02 ± 0.50 | 1.51 ± 0.46 | 1.49 ± 0.55 | 1.05 [0.57, 1.53] | 1.00 [0.52, 1.48] | 0.04 [-0.41, 0.49] |
| Arnold et al. | 2012 | 2.36 ± 0.48 | 2.03 ± 0.64 | 1.97 ± 0.68 | 0.57 [0.01, 1.14] | 0.65 [0.08, 1.22] | 0.09 [-0.47, 0.64] |
| Li et al. | 2013 | 23.50 ± 4.20 | 22.60 ± 3.70 | 21.60 ± 4.50 | 0.22 [-0.27, 0.72] | 0.43 [-0.07, 0.93] | 0.24 [-0.26, 0.74] |
| Meisel et al. | 2013 | 19.25 ± 3.70 | 12.67 ± 6.51 | 12.00 ± 5.39 | 1.20 [0.32, 2.08] | 1.51 [0.59, 2.44] | 0.11 [-0.69, 0.91] |
| Christiansen et al. | 2014 | 18.39 ± 6.84 | 14.50 ± 7.16 | 14.72 ± 5.31 | 0.54 [-0.12, 1.21] | 0.59 [-0.08, 1.25] | -0.03 [-0.69, 0.62] |
| Steiner et al. | 2014 | 80.07 ± 10.77 | 71.43 ± 10.79 | 70.06 ± 13.17 | 0.79 [0.30, 1.29] | 0.82 [0.33, 1.32] | 0.11 [-0.36, 0.59] |
| Bink et al. | 2016 | 4.63 ± 2.41 | 2.95 ± 2.63 | 2.73 ± 2.32 | 0.66 [0.21, 1.10] | 0.80 [0.35, 1.25] | 0.09 [-0.35, 0.52] |
| Duric et al. | 2017 | 15.30 ± 4.60 | 14.30 ± 3.50 | 13.90 ± 5.30 | 0.24 [-0.33, 0.81] | 0.28 [-0.29, 0.85] | 0.09 [-0.48, 0.65] |
| Geladé et al. | 2017 | 1.42 ± 0.44 | 1.04 ± 0.80 | 0.71 ± 0.68 | 0.58 [-0.06, 1.21] | 1.22 [0.53, 1.90] | 0.44 [-0.19, 1.06] |
| Hyperactivity | | | | | | | |
| Heinrich et al. | 2004 | 1.27 ± 0.52 | 0.94 ± 0.58 | 0.81 ± 0.42 | 0.58 [-0.21, 1.37] | 0.94 [0.12, 1.76] | 0.25 [-0.52, 1.02] |
| Gevensleben et al. | 2010 | 1.10 ± 0.67 | 0.79 ± 0.69 | 0.76 ± 0.68 | 0.45 [-0.00, 0.91] | 0.50 [0.04, 0.96] | 0.04 [-0.41, 0.49] |
| Arnold et al. | 2012 | 1.49 ± 0.86 | 1.20 ± 0.77 | 1.16 ± 0.69 | 0.35 [-0.21, 0.91] | 0.42 [-0.14, 0.98] | 0.05 [-0.50, 0.61] |
| Li et al. | 2013 | 18.50 ± 5.00 | 16.60 ± 4.70 | 16.00 ± 4.00 | 0.39 [-0.11, 0.88] | 0.54 [0.04, 1.05] | 0.14 [-0.36, 0.63] |
| Meisel et al. | 2013 | 13.00 ± 6.65 | 10.42 ± 6.32 | 8.50 ± 6.33 | 0.38 [-0.42, 1.19] | 0.67 [-0.16, 1.50] | 0.29 [-0.51, 1.10] |
| Christiansen et al. | 2014 | 18.78 ± 7.67 | 13.39 ± 7.53 | 13.44 ± 7.23 | 0.69 [0.02, 1.37] | 0.70 [0.03, 1.38] | -0.01 [-0.66, 0.65] |
| Steiner et al. | 2014 | 76.92 ± 13.54 | 72.73 ± 14.38 | 72.36 ± 16.34 | 0.30 [-0.18, 0.77] | 0.30 [-0.18, 0.78] | 0.02 [-0.45, 0.50] |
| Bink et al. | 2016 | 3.56 ± 2.12 | 2.49 ± 2.20 | 2.05 ± 2.22 | 0.49 [0.05, 0.93] | 0.69 [0.24, 1.14] | 0.20 [-0.24, 0.63] |
| Duric et al. | 2017 | 16.80 ± 8.00 | 9.20 ± 6.30 | 10.00 ± 9.50 | 1.04 [0.43, 1.64] | 0.76 [0.17, 1.35] | -0.10 [-0.66, 0.47] |
| Geladé et al. | 2017 | 1.30 ± 0.67 | 0.85 ± 0.71 | 0.56 ± 0.66 | 0.63 [-0.00, 1.27] | 1.09 [1.42, 1.76] | 0.41 [-0.22, 1.04] |

**Table S-6:** Control group data for inattention and hyperactivity/impulsivity domain: extracted data (M ± SD) and RevMan effect sizes
(SMD [95% confidence intervals]).

| Author | Year | Pre | Post | FU | PrePost | PreFU | PostFU |
| --- | --- | --- | --- | --- | --- | --- | --- |
| Inattention | | | | | | | |
| Gevensleben et al. | 2010 | 1.70 ± 0.46 | 1.54 ± 0.60 | 1.56 ± 0.60 | 0.29 [-0.29, 0.88] | 0.26 [-0.32, 0.84] | -0.03 [-0.61, 0.55] |
| Arnold et al. | 2012 | 2.25 ± 0.49 | 1.75 ± 0.63 | 1.95 ± 0.60 | 0.85 [-0.03, 1.73] | 0.53 [-0.33, 1.38] | -0.31 [-1.15, 0.53] |
| Li et al. | 2013 | 22.90 ± 6.10 | 23.90 ± 6.00 | 25.70 ± 4.70 | -0.16 [-0.65, 0.33] | -0.50 [-1.02, 0.01] | -0.33 [-0.83, 0.18] |
| Meisel et al. | 2013 | 19.27 ± 3.26 | 12.27 ± 4.69 | 14.09 ± 4.21 | 1.67 [0.67, 2.66] | 1.32 [0.38, 2.26] | -0.39 [-1.24, 0.45] |
| Christiansen et al. | 2014 | 20.62 ± 4.58 | 14.33 ± 5.11 | 15.10 ± 5.87 | 1.27 [0.60, 1.94] | 1.03 [0.38, 1.68] | -0.09 [-0.70, 0.51] |
| Steiner et al. CT | 2014 | 74.78 ± 9.50 | 70.21 ± 10.31 | 67.56 ± 9.05 | 0.46 [-0.03, 0.94] | 0.77 [0.28, 1.26] | 0.31 [-0.17, 0.79] |
| Steiner et al. WL |  | 76.72 ± 10.02 | 75.16 ± 10.47 | 74.58 ± 10.03 | 0.15 [-0.31, 0.61] | 0.21 [-0.25, 0.67] | 0.06 [-0.40, 0.52] |
| Bink et al. | 2016 | 5.42 ± 2.04 | 4.00 ± 2.31 | 4.05 ± 2.84 | 0.64 [-0.02, 1.29] | 0.54 [-0.11, 1.19] | -0.02 [-0.65, 0.62] |
| Duric et al. | 2017 | 17.80 ± 6.30 | 15.90 ± 6.30 | 12.10 ± 5.50 | 0.30 [-0.23, 0.82] | 0.95 [0.40, 1.51] | 0.63 [0.10, 1.17] |
| Geladé et al. MPH | 2017 | 1.34 ± 0.71 | 0.32 ± 0.77 | 0.57 ± 0.65 | 1.35 [0.67, 2.03] | 1.11 [0.46, 1.76] | -0.34 [-0.95, 0.27] |
| Geladé et al. PA |  | 1.03 ± 0.50 | 0.86 ± 0.79 | 0.73 ± 0.86 | 0.25 [-0.42, 0.93] | 0.42 [-0.26, 1.10] | 0.15 [-0.52, 0.83] |
| Hyperactivity | | | | | | | |
| Gevensleben et al. | 2010 | 1.18 ± 0.68 | 1.08 ± 0.79 | 1.00 ± 0.78 | 0.13 [-0.45, 0.71] | 0.24 [-0.34, 0.82] | 0.10 [-0.48, 0.68] |
| Arnold et al. | 2012 | 1.41 ± 0.61 | 1.15 ± 0.58 | 1.20 ± 0.59 | 0.42 [-0.43, 1.27] | 0.34 [-0.51, 1.18] | -0.08 [-0.92, 0.75] |
| Li et al. | 2013 | 18.10 ± 6.00 | 17.30 ± 6.30 | 19.20 ± 6.10 | 0.13 [-0.36, 0.62] | -0.18 [-0.68, 0.32] | -0.30 [-0.81, 0.20] |
| Meisel et al. | 2013 | 13.09 ± 6.82 | 10.45 ± 5.91 | 12.73 ± 5.52 | 0.40 [-0.45, 1.24] | 0.06 [-0.78, 0.89] | -0.38 [-1.23, 0.46] |
| Christiansen et al. | 2014 | 19.09 ± 7.71 | 13.52 ± 6.42 | 13.52 ± 7.57 | 0.77 [0.14, 1.40] | 0.72 [0.09, 1.34] | 0.00 [-0.60, 0.60] |
| Steiner et al. CT | 2014 | 72.04 ± 13.69 | 73.07 ± 15.75 | 72.19 ± 12.92 | -0.07 [-0.54, 0.41] | -0.01 [-0.49, 0.46] | 0.06 [-0.42, 0.54] |
| Steiner et al. WL |  | 77.03 ± 13.77 | 75.42 ± 14.51 | 77.16 ± 13.60 | 0.11 [-0.35, 0.57] | -0.01 [-0.47, 0.45] | -0.12 [-0.58, 0.34] |
| Bink et al. | 2016 | 2.95 ± 1.87 | 2.53 ± 2.39 | 2.53 ± 2.27 | 0.19 [-0.45, 0.83] | 0.20 [-0.44, 0.84] | 0.00 [-0.64, 0.64] |
| Duric et al. | 2017 | 18.50 ± 11.20 | 12.20 ± 10.10 | 11.40 ± 9.20 | 0.58 [0.05, 1.12] | 0.68 [0.14, 1.22] | 0.08 [-0.44, 0.61] |
| Geladé et al. MPH | 2017 | 1.03 ± 0.52 | 0.32 ± 0.70 | 0.47 ± 0.63 | 1.13 [0.47, 1.79] | 0.95 [0.31, 1.59] | -0.22 [-0.83, 0.39] |
| Geladé et al. PA |  | 0.90 ± 0.83 | 0.62 ± 0.88 | 0.48 ± 0.94 | 0.32 [-0.36, 1.00] | 0.46 [-0.22, 1.14] | 0.15 [-0.52, 0.82] |

Figure S-3: Bar graph representations of the within-group analysis standard mean deviation (SMD) findings for inattention and hyperactivity/impulsivity. Error bars represent the 95% confidence intervals. * indicates p <.05; ** indicates p < .001. Significance is only displayed for those groups that did not have significant heterogeneity.

**Table S-7**: Sensitivity analysis for within-group standard NF protocols. Standard Protocols include the results from: Heinrich et al. 2004, Gevensleben et al. 2010, Duric et al. 2012, Li et al. 2013, Meisel et al. 2013, Christiansen et al. 2014, Steiner et al. 2014, and Gelade et al. 2017.

| Within-Group Analyses |  |  |  | Effect of Intervention | | | Heterogeneity | | |
| --- | --- | --- | --- | --- | --- | --- | --- | --- | --- |
| Outcome | Trials Included | Measurement | n | SMD | 95% CI | P | Chi^2^ | I^2^ | p |
| Inattention | All | Pre-post | 10 | 0.64 | 0.45, 0.82 | <0.00001 | 9.69 | 7 | 0.38 |
|  |  | Pre-FU | 10 | 0.80 | 0.58, 1.01 | <0.00001 | 12.37 | 27 | 0.19 |
|  |  | Post-FU | 10 | 0.14 | -0.03, 0.31 | 0.11 | 2.29 | 0 | 0.99 |
|  | Standard | Pre-post | 8 | 0.65 | 0.40, 0.89 | <0.00001 | 9.63 | 27 | 0.21 |
|  |  | Pre-FU | 8 | 0.83 | 0.58, 1.12 | <0.00001 | 12.15 | 42 | 0.10 |
|  |  | Post-FU | 8 | 0.16 | -0.04, 0.36 | 0.12 | 2.17 | 0 | 0.95 |
| Hyperactivity/impulsivity | All | Pre-post | 10 | 0.50 | 0.33, 0.68 | <0.00001 | 4.86 | 0 | 0.85 |
|  |  | Pre-FU | 10 | 0.61 | 0.43, 0.79 | <0.00001 | 5.44 | 0 | 0.79 |
|  |  | Post-FU | 10 | 0.11 | -0.06, 0.28 | 0.21 | 2.27 | 0 | 0.99 |
|  | Standard | Pre-post | 8 | 0.52 | 0.32, 0.73 | <0.00001 | 4.52 | 0 | 0.72 |
|  |  | Pre-FU | 8 | 0.62 | 0.41, 0.82 | <0.00001 | 4.86 | 0 | 0.68 |
|  |  | Post-FU | 8 | 0.10 | -0.10, 0.30 | 0.33 | 2.06 | 0 | 0.96 |

**Table S-8**: Sensitivity analysis for between-group standard NF protocols. NA= non-active, Standard= only including studies with ‘standard’ NF protocols. Standard Protocols include the results from seven studies, Heinrich et al. 2004, Gevensleben et al. 2010, Duric et al. 2012, Li et al. 2013, Meisel et al. 2013, Christiansen et al. 2014, Steiner et al. 2014, and Gelade et al. 2017, two of which had two control groups.

| Between-Group Analyses | |  |  | Effect of Intervention | | | Heterogeneity | | |
| --- | --- | --- | --- | --- | --- | --- | --- | --- | --- |
| Outcome | Trials Included | Measurement | n | SMD | 95% CI | P | Chi^2^ | I^2^ | p |
| Inattention | All | Pre-post | 11 | 0.09 | -0.22, 0.40 | 0.57 | 30.60 | 67 | 0.0007 |
|  |  | Pre-FU | 11 | 0.31 | -0.01, 0.63 | 0.06 | 33.27 | 70 | 0.0002 |
|  |  | Post-FU | 11 | 0.15 | -0.07, 0.37 | 0.19 | 15.79 | 37 | 0.11 |
|  | Standard | Pre-post | 9 | 0.12 | -0.24, 0.48 | 0.51 | 28.54 | 72 | 0.0004 |
|  |  | Pre-FU | 9 | 0.33 | -0.06, 0.72 | 0.10 | 32.93 | 76 | <0.0001 |
|  |  | Post-FU | 9 | 0.13 | -0.13, 0.40 | 0.31 | 15.24 | 47 | 0.05 |
|  | NA | Pre-post | 7 | 0.38 | 0.14, 0.61 | 0.002 | 7.83 | 23 | 0.25 |
|  |  | Pre-FU | 7 | 0.57 | 0.34, 0.81 | <0.00001 | 7.48 | 20 | 0.28 |
|  |  | Post-FU | 7 | 0.17 | -0.04, 0.37 | 0.11 | 4.49 | 0 | 0.61 |
|  | NA Standard | Pre-post | 5 | 0.52 | 0.28, 0.75 | <0.0001 | 1.61 | 0 | 0.81 |
|  |  | Pre-FU | 5 | 0.57 | 0.45, 0.92 | <0.00001 | 5.68 | 0 | 0.42 |
|  |  | Post-FU | 5 | 0.15 | -0.08, 0.38 | 0.19 | 4.02 | 0 | 0.40 |
| Hyperactivity/impulsivity | All | Pre-post | 11 | 0.16 | -0.02, 0.33 | 0.07 | 5.53 | 0 | 0.85 |
|  |  | Pre-FU | 11 | 0.32 | 0.15, 0.49 | 0.0003 | 5.73 | 0 | 0.84 |
|  |  | Post-FU | 11 | 0.15 | -0.02, 0.32 | 0.08 | 7.76 | 0 | 0.65 |
|  | Standard | Pre-post | 9 | 0.15 | -0.04, 0.33 | 0.12 | 5.09 | 0 | 0.75 |
|  |  | Pre-FU | 9 | 0.31 | 0.12, 0.49 | 0.001 | 4.96 | 0 | 0.76 |
|  |  | Post-FU | 9 | 0.15 | -0.04, 0.34 | 0.11 | 7.74 | 0 | 0.46 |
|  | NA | Pre-post | 7 | 0.25 | 0.05, 0.45 | 0.02 | 0.85 | 0 | 0.99 |
|  |  | Pre-FU | 7 | 0.39 | 0.19, 0.59 | 0.0002 | 2.09 | 0 | 0.91 |
|  |  | Post-FU | 7 | 0.14 | -0.06, 0.34 | 0.18 | 2.63 | 0 | 0.85 |
|  | NA Standard | Pre-post | 5 | 0.26 | 0.03, 0.49 | 0.03 | 0.44 | 0 | 0.98 |
|  |  | Pre-FU | 5 | 0.39 | 0.16, 0.62 | 0.0008 | 1.41 | 0 | 0.84 |
|  |  | Post-FU | 5 | 0.13 | -0.10, 0.36 | 0.26 | 2.59 | 0 | 0.63 |
